# Noncanonical Activity of Tissue Inhibitor of Metalloproteinases 2 (TIMP2) Improves Cognition and Synapse Density in Aging

**DOI:** 10.1523/ENEURO.0031-23.2023

**Published:** 2023-06-12

**Authors:** Rachel Britton, Tristan Wasley, Reema Harish, Charles Holz, John Hall, Dennis C. Yee, Jody Melton Witt, Elizabeth A. Booth, Steven Braithwaite, Eva Czirr, Meghan Kerrisk Campbell

**Affiliations:** 1 Alkahest, Inc., San Carlos, CA 94070; 2 Grifols Diagnostic Solutions, Inc., Emeryville, CA 94608

**Keywords:** *cfos*, cognition, MMP, mouse, synapse, TIMP2

## Abstract

Peripheral administration of tissue inhibitor of metalloproteinases 2 (TIMP2), a protein inhibitor of matrix metalloproteinases (MMPs), has previously been shown to have beneficial effects on cognition and neurons in aged mice. Here, to better understand the potential of recombinant TIMP2 proteins, an IgG4Fc fusion protein (TIMP2-hIgG4) was developed to extend the plasma half-life of TIMP2. Following one month of administration of TIMP2 or TIMP2-hIgG4 via intraperitoneal injections, 23-month-old male C57BL/6J mice showed improved hippocampal-dependent memory in a Y-maze, increased hippocampal *cfos* gene expression, and increased excitatory synapse density in the CA1 and dentate gyrus (DG) of the hippocampus. Thus, fusion to hIgG4 extended the half-life of TIMP2 while retaining the beneficial cognitive and neuronal effects. Moreover, it retained its ability to cross the blood-brain barrier. To deepen the mechanistic understanding of the beneficial function of TIMP2 on neuronal activity and cognition, a TIMP2 construct lacking MMP inhibitory activity, Ala-TIMP2, was generated, which provides steric hindrance that prevents inhibition of MMPs by the TIMP2 protein while still allowing MMP binding. A comprehensive assessment of the MMP inhibitory and binding capacity of these engineered proteins is outlined. Surprisingly, MMP inhibition by TIMP2 was not essential for its beneficial effects on cognition and neuronal function. These findings both confirm previously published research, expand on the potential mechanism for the beneficial effects of TIMP2, and provide important details for a therapeutic path forward for TIMP2 recombinant proteins in aging-related cognitive decline.

## Significance Statement

We identify a novel mechanism for tissue inhibitor of metalloproteinases 2 (TIMP2) in age-related cognitive decline and provide evidence for a fusion protein with an extended plasma half-life to improve cognition and neuronal connectivity. While TIMP2 has been previously shown to be beneficial for the aging CNS, this study demonstrates that its benefit is unlikely to be directly mediated via matrix metalloproteinase (MMP) inhibition. These considerations provide important details for the potential therapeutic utility of TIMP2 recombinant proteins to reverse age-related cognitive decline.

## Introduction

Aging is the number one risk factor for developing diseases of the body and of the mind, such as Alzheimer’s disease. In aged mice, many of the effects of aging were attenuated in studies using heterochronic parabiosis (Villeda et al., 2011), and further confirmed with direct administration of young plasma (Villeda et al., 2014). Furthermore, human umbilical cord plasma has been shown to significantly improve behavior, long-term potentiation (LTP), and c-Fos protein levels in aged immunodeficient ‘NOD-SCID’ (NSG) mice (Castellano et al., 2017). One of the active proteinaceous factors identified in cord plasma was tissue inhibitor of metalloproteinases 2 (TIMP2). Injection of recombinant TIMP2 into aged wild-type (WT) C57BL/6 mice produced similar effects to dosing cord plasma in aged NSG mice, including improved behavioral outcomes in Barnes maze and contextual fear conditioning, enhanced LTP, and increased c-Fos staining in the dentate gyrus (DG) of the hippocampus (Castellano et al., 2017). Lower TIMP2 concentrations are associated with multiple human conditions: in cerebral spinal fluid its level negatively correlates with microbleeds in Alzheimer’s disease (Duits et al., 2015), in plasma lower TIMP2 is found in patients with frontotemporal dementia (Lorenzl et al., 2008), and in blood TIMP2 negatively correlates with cognitive deficits in recurrent depressive disorder (Bobińska et al., 2016). These data suggest that loss of TIMP2 is associated with cognitive deficits and that supplementation of TIMP2 may be a beneficial therapeutic strategy for improving CNS function.

Canonically, TIMP2 is known to inhibit matrix metalloproteinases (MMPs), which regulate extracellular matrix degradation. However, TIMP2 also has MMP-independent functions in proliferation (Hoegy et al., 2001; Seo et al., 2003, 2008, 2011; Pérez-Martínez and Jaworski, 2005; Fernandez et al., 2010; H. J. Kim et al., 2014), cell migration (Terasaki et al., 2003; Oh et al., 2004), endothelial cell permeability (S. H. Kim et al., 2012), and tube formation (H. J. Kim et al., 2014) *in vitro*, as well as angiogenesis (Seo et al., 2003, 2011) and vascular permeability (S. H. Kim et al., 2012) *in vivo*. Within the CNS, TIMP2 promotes neuronal differentiation and neurite outgrowth through an MMP-independent mechanism (Pérez-Martínez and Jaworski, 2005), suggesting that the beneficial cognitive effects with TIMP2 treatment may occur through its MMP-independent functions. Recently, TIMP2 was identified to be highly expressed by neurons in the hippocampus, and loss of TIMP2 leads to impairments in neurogenesis, dendritic spines, and hippocampus-dependent memory (Ferreira et al., 2022).

Here, to better understand the therapeutic potential of recombinant TIMP2 proteins, an IgG4Fc fusion protein was generated to extend the plasma half-life of TIMP2. Additionally, to deepen the mechanistic understanding of the beneficial function of TIMP2 on neuronal activity and cognition, a TIMP2 construct lacking MMP inhibitory activity, Ala-TIMP2, was generated, which provides steric hindrance that prevents inhibition of MMPs by the TIMP2 protein while still allowing MMP binding (Wingfield et al., 1999). We provide a comprehensive assessment of the MMP inhibitory and binding impacts of these engineered proteins. Furthermore, MMP inhibition by TIMP2 was not essential for its beneficial effects on cognition and neuronal function. These findings confirm previously published research (Castellano et al., 2017), expand on the potential mechanism for the beneficial effects of TIMP2, and provide important details for a therapeutic path forward for TIMP2 recombinant proteins in aging-related cognitive decline.

## Materials and Methods

### Animals

All animal handling and use was in accordance with Institutional Animal Care and Use Committee approved protocols. Male C57BL/6J mice were obtained from The Jackson Laboratory and shipped before the start of each study. All animals were acclimated in-house for at least two weeks before the start of the experiments. Upon arrival all mice were single housed with a unique identification number at standard temperature (22 ± 1°C) and in a light-controlled environment (lights on from 7 A.M. to 7 P.M.) with *ad libitum* access to food and water. To homogenize treatment groups, mice were assessed on nesting performance and memory in Y-maze before chronic protein administration. Mouse weight, nesting performance, cognitive performance, total distance traveled in Y-maze, and average velocity in Y-maze were used to evenly group animals between vehicle and treatment groups. Seven cohorts of mice were used for experiments outlined in Table 1.

**Table 1:** Description of mouse cohorts

Cohort	Treatment groups	Age at start	Dosage	Dosing length	Figures
Cohort 1	TIMP2, TIMP2-hIgG4	6.5 M	250 μg/kg	Single	Figure 1*A* (PK)
Cohort 2	TIMP2, Ala-TIMP2	2 M	250 μg/kg	Single	Figure 6*B* (PK)
Cohort 3	Vehicle, TIMP2, TIMP2-hIgG4	23 M	250 μg/kg	4 weeks	Figures 1*C–H* (behavior and endogenous protein), 2A–C,F,G (immediate early genes and neurogenesis), 3A–I (synapses and microglia); Tables 2, 3 (gene expression)
Cohort 4	Vehicle, TIMP2, Ala-TIMP2	21.5 M	250 μg/kg	4 weeks	Figure 6*A* (behavior)
Cohort 5	Vehicle, TIMP2, Ala-TIMP2	21.7 M	250 μg/kg	4 weeks	Figure 6*C–E* (synapses)
Cohort 6	Vehicle, TIMP2	18 M	50 μg/kg	1 week	Figure 2*D,E* (iDISCO c-Fos); Movie 1
Cohort 7	Vehicle, TIMP2, TIMP2-hIgG4	22 M	1 mg/kg	Single	Figure 3*J* (brain penetrance)

Table provides details on each mouse cohort used and corresponding figures. M, months; PK, pharmacokinetics.

Movie 1. Subtle changes in c-Fos across the entire brain following TIMP2 treatment. C-Fos across the entire brain was detected using a 3D imaging of solvent-cleared organs (iDISCO) procedure. Movie was generated from individual images that represent the average of the difference between vehicle-treated and TIMP2-treated mice. Red color represents increased c-Fos in TIMP2 treatment relative to vehicle and green color represents decreased c-Fos in TIMP2 treatment relative to vehicle. 10.1523/ENEURO.0031-23.2023.video.1

### Key resources table

Details on key resources used are provided in Table 2.

**Table 2:** Key resources

Reagent type (species) or resource	Designation	Source or reference	Identifiers	Additional information
Strain, strain background (*Mus musculus*)	C57BL/6J	The Jackson Laboratory	Stock #000664 RRID: IMSR_JAX:000664	
Cell line	Human Embryonic Kidney 293-6E	Thermo Fisher	Catalog #A14527	For work done at Grifols and Proteos
Peptide, recombinant protein	Human TIMP2(1–220)	Grifols Diagnostic Solutions	N/A	
Peptide, recombinant protein	Human TIMP2(1-220)_human IgG4Fc	Grifols Diagnostic Solutions	N/A	TIMP2-hIgG4
Peptide, recombinant protein	Human TIMP2(1–26)_Ala_TIMP2(27–220)	Grifols Diagnostic Solutions	N/A	Ala-TIMP2
Peptide, recombinant protein	Recombinant Mouse MMP-2 (carrier-free)	BioLegend	Catalog #554404	Pro-enzyme Ms-pro-MMP2
Peptide, recombinant protein	Recombinant Mouse MMP-3 (carrier-free)	BioLegend	Catalog #552704	Pro-enzyme Ms-pro-MMP3
Peptide, recombinant protein	Recombinant Mouse MMP-9 (carrier-free)	BioLegend	Catalog #755204	Pro-enzyme Ms-pro-MMP9
Peptide, recombinant protein	Recombinant Human MMP-2 (carrier-free, pro-enzyme)	R&D Systems	Catalog #902-MP-010	Pro-enzyme Hu-pro-MMP2.0
Peptide, recombinant protein	Recombinant Human MMP-2 (carrier-free)	BioLegend	Catalog #554304	Pro-enzyme Hu-pro-MMP2.1
Peptide, recombinant protein	Recombinant Human MMP-3 (carrier-free)	BioLegend	Catalog #594704	Pro-enzyme Hu-pro-MMP3
Peptide, recombinant protein	Recombinant Human MMP-9 (carrier-free)	BioLegend	Catalog #550504	Pro-enzyme Hu-pro-MMP9
Peptide, recombinant protein	Recombinant Human MMP-2 (pro-enzyme)	AnaSpec	Catalog #AS-72005	Pro-enzyme Hu-pro-MMP2.2
Peptide, recombinant protein	Recombinant Human MMP-3 (catalytic domain)	AnaSpec	Catalog #AS-72006	Catalytic domain (active) Hu-CD-MMP3
Peptide, recombinant protein	Recombinant Human MMP-9 (catalytic domain)	AnaSpec	Catalog #AS-55576-1	Catalytic domain (active) Hu-CD-MMP9
Antibody	Anti-Doublecortin (guinea pig polyclonal)	Millipore	Catalog #AB2253 RRID: AB_1586992	IHC 1:2000
Antibody	Anti-EGR1, clone 15F7 (rabbit monoclonal)	Cell Signaling Technology	Catalog #4153 RRID: AB_2097038	IHC 1:2000
Antibody	Anti-CD68, clone FA-11 (rat monoclonal)	Bio-Rad	Catalog #MCA1957 RRID: AB_322219	IHC 1:1000
Antibody	Anti-Iba1 (rabbit polyclonal)	FUJIFILM Wako Pure Chemical Corporation	Catalog #019-19741 RRID: AB_839504	IHC 1:2500
Antibody	Anti-Synapsin-1/2 (chicken polyclonal)	Synaptic Systems	Catalog #106006 RRID: AB_262240	IHC 1:1000
Antibody	Anti-PSD-95, clone D27E11 (rabbit monoclonal)	Cell Signaling Technology	Catalog #3450 RRID: AB_2292883	IHC 1:250
Antibody	Anti-Homer1 (rabbit polyclonal)	Synaptic Systems	Catalog #160003 RRID: AB_887730	IHC 1:500
Antibody	Anti-Gephyrin	Synaptic Systems	Catalog #147018 RRID: AB_2651176	IHC 1:500
Antibody	Alexa 555 or 647 secondaries	Invitrogen		IHC 1:300
Antibody	Anti-c-Fos, clone 9F6 (rabbit monoclonal)	Cell Signaling Technology	Catalog #2250 RRID: AB_2247211	IHC 1:1000 iDISCO 1:1000
Antibody	Biotinylated anti-guinea pig IgG (goat)	Vector Laboratories	Catalog #BA-7000 RRID: AB_2336132	IHC 1:300
Other	Hoechst	Invitrogen	Catalog #H3570	IHC 1:10,000
Other	Prolong Gold Antifade Mountant	Invitrogen	Catalog #P36934	
Other	Series S Sensor Chip CM5	Cytiva	Catalog #29149603	
Other	Streptavadin Octet Tips	Sartorius	Catalog #18-0009	SA
Other	Protein A Octet Tips	Sartorius	Catalog #18–0004	ProA
Other	4–15% Criterion TGX Stain Free Midi Protein Gel, 26-well	Bio-Rad	Catalog #6578085	
Other	Hitrap SP Sepharose HP Column	Cytiva	Catalog #17115201	For work done at Grifols
Other	SP-Sepharose Fast Flow Resin	Cytiva	Catalog #17072901	For work done at Proteos
Other	HiTrap MabSelect SuRe	Cytiva	Catalog #11003495	For work done at Grifols
Other	Protein A Praesto AC	Purolite	Catalog #PR00200-310	For work done at Proteos
Other	HiLoad 16/600 Superdex 75-pg Size Exclusion Column	Cytiva	Catalog #28989333	For work done at Grifols
Other	Superdex 75 Size Exclusion Column	Cytiva	Catalog # dependent on size or quantity ordered	For work done at Proteos
Other	PEIpro, linear	Polyplus	Catalog #115-01L	For work done at Proteos
Chemical compound	3,3′-Diaminobenzidine tetrahydrochloride (DAB)	Sigma-Aldrich	Catalog #D5905	
Chemical compound	Citrisolv Clearing Agent	Decon Labs	Catalog #22-143-975	
Chemical compound	Cytoseal	Thermo Fisher	Catalog #8310-4	
Chemical compound	Tissue Extraction Reagent I	Thermo Fisher	Catalog #FNN0071	
Chemical compound, drug	2,2,2-tribromoethanol (Avertin)	Sigma-Aldrich	Catalog #T48402-25G	1.61 g/ml stock diluted 1:40 in sterile saline
Chemical compound	Corning Dulbecco’s PBS (DPBS)	Spectrum Chemical	Catalog #21-030-CM	For dilution of TIMP2 constructs
Chemical compound	Paraformaldehyde (32% stock)	Electron Microscopy Sciences	Catalog #15714S	4% working solution made in PBS
Chemical compound	Sucrose	Fisher Scientific	Catalog #S5-3	30% w/v working solution made in PBS
Chemical compound	Ethylene glycol	Fisher Scientific	Catalog #E178-4	
Chemical compound	Glycerol	Sigma-Aldrich	Catalog #G5516	
Chemical compound	Ethylenediaminetetraacetic acid (EDTA)	Boston BioProducts	Catalog #BM-711	
Chemical compound	HBS-P+, 10× concentrated; 0.1 m HEPES, 1.5 m NaCl, 0.5 v/v Surfactant P20, pH 7.4	Cytiva	Catalog #BR100671	
Chemical compound	BIA normalizing solution (70% glycerol)	Cytiva	Catalog #29207950	
Chemical compound	10 mm glycine-HCl, pH 2.5	Cytiva	Catalog #BR100356	Glycine 2.5
Chemical compound	50 mm sodium hydroxide	Cytiva	Catalog #BR100358	NaOH 50
Chemical compound	10 mm sodium acetate, pH 5.0	Cytiva	Catalog #BR100351	Acetate 5.0
Chemical compound	Bovine serum albumin, heat shock fraction, suitable for RIA, pH 5.2, ≥96%	Sigma-Aldrich	Catalog #A7888-100g	BSA
Chemical compound	PBS 20×, pH 7.5, Ultra Pure	VWR	Catalog #E703-1L	For work done at Grifols Diluted to 1× in Milli-Q H_2_O
Chemical compound	10× PBS, pH 7.4	Corning	Catalog #46-013-CM	For work done at Proteos Diluted to 1× in Milli-Q H_2_O
Chemical compound	100% Polyoxyethyenesorbitan monolaurate	Sigma-Aldrich	Catalog #P-7949	Tween 20
Chemical compound	1 m Tris-HCl, pH 8.0	Teknova	Catalog #T1080	For work done at Grifols
Chemical compound	1 m Tris-HCl, pH 8.0	Corning	Catalog #46-031-CM	For work done at Proteos
Chemical compound	5 m NaCl	Quality Biologicalalal	Catalog #351-036-491	For work done at Grifols Diluted to 1 m in Milli-Q H_2_O
Chemical compound	5 m NaCl	Corning	Catalog #46-032-CV	For work done at Proteos
Chemical compound	1 m NaOAc, pH 5.0	Teknova	Catalog #S0391	For work done at Grifols Diluted to 2 mm in Milli-Q H_2_O
Chemical compound	NaOAc	JT Baker	Catalog #3470	For work done at Proteos Working stock: 25 mm NaOAc, pH 5.0
Chemical compound	EXPI293 Expression Media	Thermo Fisher	Catalog #A1435102	For work done at Grifols
Chemical compound	F17 supplemented with 0.1% Pluronic F-68, 4 mm GlutaMAX, 25 μg/ml G418	Life Technologies	Catalog # dependent on size or quantity ordered	For work done at Proteos
Commercial assay or kit	Vectastain ABC kit	Vector Laboratories	Catalog #PK-4000	
Commercial assay or kit	Mouse TIMP-2 DuoSet ELISA	R&D Systems	Catalog #DY6304	
Commercial assay or kit	Human TIMP-2 DuoSet ELISA	R&D Systems	Catalog #DY971	
Commercial assay or kit	DuoSet ELISA Ancillary Reagent Kit 2	R&D Systems	Catalog #DY008	
Commercial assay or kit	Mouse MMP2 ELISA kit	Sigma-Aldrich	Catalog #RAB0366	
Commercial assay or kit	SensoLyte 520 MMP-2 Assay kit, Fluorimetric	AnaSpec	Catalog #AS-71151	
Commercial assay or kit	SensoLyte 520 MMP-3 Assay kit, Fluorimetric	AnaSpec	Catalog #AS-71152	
Commercial assay or kit	SensoLyte 520 MMP-9 Assay kit, Fluorimetric	AnaSpec	Catalog #AS-71155	
Commercial assay or kit	RNeasy Mini kit	QIAGEN	Catalog #74106	
Commercial assay or kit	Superscript III First-Strand Synthesis SuperMix kit	Invitrogen	Catalog #11752050	
Commercial assay or kit	Applied Biosystems SYBR Green PCR Master Mix	Thermo Fisher	Catalog #43-091-55	
Commercial assay or kit	Applied Biosystems TaqMan Multiplex Master Mix	Thermo Fisher	Catalog #44-842-63	
Commercial assay or kit	Amine Coupling kit	Cytiva	Catalog #BR100050	
Commercial assay or kit	EZ-Link NHS-PEG4-Biotin, No-Weigh Format Biotinlyation kit	Thermo Fisher	Catalog #A39259	
Commercial assay or kit	ExpiFectamine 293 Transfection kit	Thermo Fisher	Catalog #A14525	
Sequence-based reagent	Mouse *Dcx* (DCX) qPCR primers	Thermo Fisher	Catalog #4331182 Assay ID: Mm00438400_m1	
Sequence-based reagent	Mouse *Tubb3* (β-tubulin III) qPCR primers	Thermo Fisher	Catalog #4331182 Assay ID: Mm00727586_s1	
Sequence-based reagent	Mouse *Syn1* (Synapsin-1) qPCR primers	Integrated DNA Technologies, Inc		GGAAGGGATCACATTATTGAGG/TGCTTGTCTTCATCCTGGTG
Sequence-based reagent	Mouse *Dlg4* (PSD-95) qPCR primers	Integrated DNA Technologies, Inc		CGCTACCAAGATGAAGACACG/CAATCACAGGGGGAGAATTG
Sequence-based reagent	Mouse *Gria1* (GluR1) qPCR primers	Thermo Fisher	Catalog #4331182 Assay ID: Mm00433753_m1	
Sequence-based reagent	Mouse *Grin2a* (GluN2A) qPCR primers	Integrated DNA Technologies, Inc		TGATGAACCGCACTGACCCTA/GGAAGAACGTGGATGTCGGA
Sequence-based reagent	Mouse *Slc2a1* (vGLUT1) qPCR primers	Integrated DNA Technologies, Inc		CCGGGCCTTGACCTTAGC/CCTCGAGCCGCTGAATTAAT
Sequence-based reagent	Mouse *Gad1* (GAD1) qPCR primers	Integrated DNA Technologies, Inc		CCTTCGCCTGCAACCTCCTCGAAC/GCGCAGTTTGCTCCTCCCCGTTC TT
Sequence-based reagent	Mouse c*fos* (c-Fos) qPCR primers	Thermo Fisher	Catalog #4331182 Assay ID: Mm00487425_m1	
Sequence-based reagent	Mouse *Creb1* (CREB1) qPCR primers	Thermo Fisher	Catalog #4331182 Assay ID: Mm00501607_m1	
Sequence-based reagent	Mouse *Egr1* (EGR1) qPCR primers	Thermo Fisher	Catalog #4331182 Assay ID: Mm00656724_m1	
Sequence-based reagent	Mouse *Il1a* (IL-1α) qPCR primers	Thermo Fisher	Catalog #4331182 Assay ID: Mm00439620_m1	
Sequence-based reagent	Mouse *Il1b* (IL-1β) qPCR primers	Thermo Fisher	Catalog #4331182 Assay ID: Mm00434228_m1	
Sequence-based reagent	Mouse *Il6* (IL-6) qPCR primers	Thermo Fisher	Catalog #4331182 Assay ID: Mm00446190_m1	
Sequence-based reagent	Mouse *Ccl11* (Eotaxin) qPCR primers	Thermo Fisher	Catalog #4331182 Assay ID: Mm00441238_m1	
Sequence-based reagent	Mouse *Nfkb* (NFκB) qPCR primers	Thermo Fisher	Catalog #4331182 Assay ID: Mm00476361_m1	
Sequence-based reagent	Mouse *Tnfa* (TNFα) qPCR primers	Thermo Fisher	Catalog #4331182 Assay ID: Mm00443258_m1	
Sequence-based reagent	Mouse *Cd68* (CD68) qPCR primers	Thermo Fisher	Catalog #4331182 Assay ID: Mm03047343_m1	
Sequence-based reagent	Mouse *Iba1* (Iba1) qPCR primers	Thermo Fisher	Catalog #4331182 Assay ID: Mm00479862_g1	
Sequence-based reagent	Mouse *Cd11b* (CD11b) qPCR primers	Thermo Fisher	Catalog #4331182 Assay ID: Mm00434455_m1	
Sequence-based reagent	Mouse *Aqp4* (AQP4) qPCR primers	Thermo Fisher	Catalog #4331182 Assay ID: Mm00802131_m1	
Sequence-based reagent	Mouse *Gfap* (GFAP) qPCR primers	Thermo Fisher	Catalog #4331182 Assay ID: Mm01253033_m1	
Sequence-based reagent	Mouse *Ggta1* (GGTA1) qPCR primers	Thermo Fisher	Catalog #4331182 Assay ID: Mm01333302_m1	
Software, algorithm	ANY-Maze	Stoelting Co	RRID: SCR_014289	
Software, algorithm	Zen	Zeiss	Zen Blue 2.5 RRID: SCR_013672	
Software, algorithm	Image-Pro	Media Cybernetics, Inc	Image-Pro 9.2 RRID: SCR_016879	
Software, algorithm	SynapseCounter (ImageJ plugin)		https://github.com/SynPuCo/SynapseCounter	
Software, algorithm	QuantStudio	Applied Biosystems	QuantStudio 6 RRID: SCR_020239	
Software, algorithm	GraphPad Prism	GraphPad Software, Inc	GraphPad Prism 8 RRID: SCR_002798	
Software, algorithm	Image Lab	Bio-Rad	Image Lab 6.0.1 RRID: SCR_014210	
Software, algorithm	Astra	Wyatt Technology	Astra 7.3.2 RRID: SCR_016255	
Software, algorithm	Unicorn	Cytiva	Unicorn 7.7 RRID: SCR_019958	
Software, algorithm	Octet Analysis Studio	ForteBio/Sartorius	Octet Analysis Studio 12.2.2.26	
Software, algorithm	Masshunter Workstation Software	Agilent Technologies	Masshunter 9.0.9044.1 SP1	
Software, algorithm	EndoScan-V	Charles River	EndoScan-V version 6.0.2	
Software, algorithm	Biacore T200 Control and Evaluation Software	Cytiva	Biacore T200 Software 3.2.1 RRID: SCR_019718	

Table provides a description of the key resources used and manufacturing product numbers. N/A, not applicable.

### Proteins

Recombinant human TIMP2 protein, human TIMP2-IgG4Fc fusion protein, and human Ala-TIMP2 protein were produced by both Proteos and Grifols Diagnostic Solutions. At Proteos, cDNA encoding all TIMP2 protein constructs were expressed by pTT5 vector and transfected into Human Embryonic Kidney 293-6E cells (A14527, Thermo Fisher Scientific) using linear PEIpro (115-01L, PolyPlus) at a 1:1 (w/v) DNA:PEI ratio and cultures were grown at 37°C and harvested 4 d after transfection. At Grifols Diagnostic Solutions, cDNA encoding all TIMP2 protein constructs were expressed by pCMVIII vector and transfected into Expi293 cells (A14527, Thermo Fisher Scientific) using Expifectamine (A14525, Fisher Scientific) under standard conditions and cultures were grown at 37.5°C and harvested 4 d after transfection. Downstream purifications at Proteos and Grifols Diagnostic Solutions were performed similarly as follows. Recombinant human TIMP2 proteins were purified by SP Sepharose resin (Proteos: 17072901, Cytiva; Grifols: 17115201, Cytiva) equilibrated with 25 mm NaOAc, pH 5.0 (23°C; Proteos: 3470, JT Baker; Grifols: S0391, Teknova) using an AKTA system (Cytiva). An initial 2 CV (Proteos) or 5 CV (Grifols) wash in equilibration buffer was performed followed by a 0–1000 mm NaCl (Proteos: 46-032-CV, Corning; Grifols: 351-036-491, Quality Biological) gradient in equilibration buffer. Fractions were identified and pooled by SDS-PAGE (6578085, Bio-Rad). Post-ion exchange chromatography pools of recombinant human TIMP2 were concentrated and then injected onto a Superdex 75 size-exclusion chromatography (SEC) column (Proteos: Size-dependent, Cytiva; Grifols: 28989333, Cytiva) with a final destination buffer of 1× PBS, pH 7.4 (23°C; Proteos: 46–013-CM, Corning; Grifols: E703-1L, VWR). Fractions to pool were identified by SDS-PAGE (6578085, Bio-Rad). Recombinant human TIMP2-hIgG4 proteins were purified by Protein A resin (Proteos: PR00200-310, Purolite; Grifols: 11003495, Cytiva) equilibrated in 1× PBS, pH 7.4 (23°C; Proteos: 46-013-CM, Corning; Grifols: E703-1L, VWR) using an AKTA system. An initial 20 CV wash in equilibration buffer was performed followed by an elution phase in 30 mm NaOAc, pH 3.6 (23°C; Proteos: 9508, JT Baker; Grifols: S0391, Teknova). Elution fractions were neutralized using a 1:10 volume of 1 m Tris, pH 8.0 (23°C; Proteos: 46-031-CM, Corning; Grifols: T1080, Teknova). Fractions were identified and pooled by SDS-PAGE (6578085, Bio-Rad).

All proteins were characterized for identity (intact mass), purity (SDS-PAGE), oligomerization and aggregation (SEC-MALS), concentration (A280), and endotoxin (LAL assay; Table 3). Intact mass was confirmed using an Agilent 6530B QTOF with PLRP-S column (Agilent Technologies); final proteins were confirmed within 100 ppm of the expected mass using the Masshunter Software (Agilent Technologies). Concentration was determined using A280 absorbance values with reference blanked sample with theoretical extinction coefficient. Purity was assessed by loading 1–3 ng in triplicate on 4–15% Criterion SDS-PAGE (6578085, Bio-Rad) or 4–20% TGX Criterion SDS-PAGE (5678094, Bio-Rad) and performing densitometry measurements using Image Lab software (Bio-Rad); purity was >85% for all samples. Oligomerization and aggregation were determined using SEC-MALS with two WTC-030S5 (Wyatt Technology) SEC columns in series on a DAWN HELEOS II (Wyatt Technology) MALS and DLS instrument. Refractive index data were collected by Optilab T-rEX (Wyatt Technology) and UV absorbance data were collected by the 1100 series MWD G1365B (Agilent Technologies) detector. Software for SEC-MALS was controlled by Astra (Wyatt Technology). Aggregation of final sample was <10% for all samples. All endotoxin LAL assays were below detection limit of 0.05 EU/ml when evaluated by the Endosafe nextgen-MCS (Charles River) using the EndoScan-V software (Charles River).

**Table 3:** Characterization of TIMP2 protein constructs

Construct	Identity (intact mass)	Concentration (mg/ml; A280)	Purity (%; R/NR, SDS-PAGE)	Aggregation (SEC-MALS)	Oligomerization (SEC-MALS)	Endotoxin (EU/ml; LAL)	
TIMP2	Confirmed	1.57	82/100	None detected	Monomer	<0.1
TIMP2-hIgG4	Confirmed	1.89	100/85	<5%	Dimer	<0.1
Ala-TIMP2	Confirmed	2.47	75–81/100	None detected	Monomer	<0.1

All recombinant TIMP2 protein constructs were characterized for identity (intact mass), concentration [A280, adjusted with calculated extinction coefficient: TIMP2, Ala-TIMP2 Abs 0.1% (=1 g/l) = 1.525; TIMP2-hIgG4 Abs 0.1% (= 1 g/l) = 1.408)], purity (SDS-PAGE), oligomerization and aggregation (SEC-MALS), and endotoxin (LAL assay) characterization. “/” in Purity column used to separate Reduced and Nonreduced percentage values, not division. R, reducing; NR, non-reducing; EU, endotoxin unit; LAL, limulus amebocyte lysate; SEC, size exclusion chromatography; MALS, multi angle light scattering.

### Protein administration

All TIMP2 proteins were diluted in sterile DPBS (21-030-CM, Spectrum Chemical) and dosed intraperitoneally at 250 μg/kg, unless otherwise stated. For chronic experiments, mice were dosed daily for four weeks. Mice dosed with DPBS vehicle, TIMP2, or Ala-TIMP2 received test compound daily, while mice receiving TIMP2-hIgG4 received test compound every third day and DPBS on the days they were not on active treatment. For the acute brain penetrance experiment, mice were given a single 1 mg/kg dose intraperitoneally. For the iDISCO c-Fos analysis, mice were dosed daily with 0.9% saline or TIMP2 at 50 μg/kg diluted in 0.9% saline for 7 d.

### Behavior

#### Nesting

Assessment of nesting performance was based on a published protocol (Deacon, 2006). Mice were placed in a clean home cage and given two nestlets in the evening toward the end of their light cycle. The next morning (16 h later), nests were scored by a blinded experimenter on a scale of 1–5, with 5 being the most dome-like complete nest.

#### Y-maze

A Y-shaped apparatus was constructed with Extruded PVC (Komatex). Each arm was 15 inches long and 3 inches wide with 6 inches tall walls. Unique cues in the form of black shapes were adhered to the walls at the ends of two of the arms, while the third arm was un-cued and designated as the starting point for the mice. Mice were habituated to a dimly lit room for at least 30 min before the start of training. First, mice were individually placed in the starting arm and allowed to explore only one of the other two arms (familiar arm) for 5 min; the second arm (novel arm) was blocked off with an acrylic plastic wall identical to that of the rest of the apparatus. After 2.5 h, each mouse was then returned to the maze with all arms now open to explore for 5 min. All movements were recorded and tracked for analysis using ANY-Maze Software (Stoelting Co). The number of entries into and the time spent in each of the two arms, familiar and novel, were measured. The total distance and velocity were also measured for the duration of the test. After each trial, the maze was wiped down thoroughly with 70% ethanol. The experimenter was blinded to treatment while performing and analyzing the experiment.

### Histology

Mice were anesthetized with 2,2,2-tribromoethanol (Avertin, T48402-25G, Sigma-Aldrich) and subsequently perfused with 0.9% saline transcardially. The brains were dissected and cut sagittally in two even halves. One half was snap frozen in dry ice for protein and RNA analysis, and the other was fixed in 4% paraformaldehyde (PFA; 15714S, Electron Microscopy Sciences) in PBS for use in immunohistochemistry. After 2 d of fixation, the hemibrains were transferred to a 30% sucrose (S5-3, Fisher Scientific) in PBS solution and then changed again after 1 d. Hemibrains were sectioned coronally at 30 μm on a microtome at −22°C. Brain slices were collected sequentially into 12 tubes, so that every 12th section of the hippocampus was represented in a given tube. Brain sections were stored in cryoprotectant media composed of 30% ethylene glycol (E178-4, Fisher Scientific) and 30% glycerol (G5516, Sigma-Aldrich) in a sodium phosphate solution at −20°C until needed for staining.

For fluorescent microscopy, blocking was done on free floating sections in the appropriate serum at 10% in PBS-Triton X-100 0.5% (215680010, ACROS Organics), unless otherwise noted. Primary antibodies were incubated overnight at 4°C. The appropriate fluorescent secondary antibodies (Invitrogen) were applied the next day at a concentration of 1:300 for 1 h at room temperature followed by Hoechst (H3570, Invitrogen) at a concentration of 1:10,000 for 10 min. Prolong Gold Antifade Mountant (P36934, Invitrogen) was used to coverslip the slides.

To stain for doublecortin (DCX) and early growth response 1 (EGR1), PBS with 1% Triton X-100 was used for all blocking, washing, and antibody steps. DCX antibody (AB2253, lot #3128122, Millipore) was used at a concentration of 1:2000 and stained with EGR1 antibody (4153, Cell Signaling Technology) at a concentration of 1:2000. DCX-positive cells in the blades of the dentate gyrus (DG) were counted live at 20× magnification on a Leica DM5500 B Upright Microscope by a single experimenter blinded to treatment. DCX/EGR1 images were acquired using the Axio Scan.Z1 (Zeiss) at 20×, and then EGR1 was quantified using percent thresholded area of the entire hippocampus region using Image-Pro 9.2 software (Media Cybernetics) by a single experimenter blinded to treatment.

CD68 antibody (MCA1957, Bio-Rad) was used at a concentration of 1:1000 and stained together with ionized calcium binding adaptor molecule 1 (Iba1) antibody (019-19741, Wako Chemicals), used at 1:2500. CD68/Iba1 images were acquired using the Axio Scan.Z1 (Zeiss) at 20×. Images were quantified using percent thresholded area of the entire hippocampus region using Image-Pro 9.2 software (Media Cybernetics) by a single experimenter blinded to treatment.

To stain for excitatory and inhibitory synapses, sections were blocked in 10% goat serum with PBS and 1% Triton X-100 for 1 h. For all synaptic stains, synapsin-1/2 antibody (106006, Synaptic Systems) at 1:1000 was used to stain the presynapse. For the excitatory postsynaptic marker, either postsynaptic density protein-95 (PSD-95) antibody (3450, Cell Signaling Technology) at 1:250 or homer1 antibody (160003, Synaptic Systems) at 1:500 was used. For the inhibitory postsynaptic marker, gephyrin antibody (147018, Synaptic Systems) at 1:500 was used. Primary antibodies were incubated overnight at 4°C in 3% goat serum in PBS with 0.5% Triton X-100. Ten z-stack (0.18-μm step size) images in the stratum radiatum of the CA1 and hilus of the DG were acquired using a Zeiss LSM800 with Airyscan at 63×, Airyscan processed using Zen Blue 2.5 (Zeiss), and then quantified using the ImageJ macro SynapseCounter (https://github.com/SynPuCo/SynapseCounter) to measure presynaptic Synapsin-1/2 puncta; postsynaptic PSD-95, Homer1, or Gephyrin puncta; and juxtaposed signal for synapses.

For light microscopy, blocking was done on free floating sections in the appropriate serum at 10% in PBS-Triton X-100 0.5%. C-Fos antibody (2250, Cell Signaling Technology) was used at a concentration of 1:1000 and incubated overnight at 4°C. Biotinylated anti-guinea pig antibody (BA-7000, Vector Laboratories) was applied the next day at a concentration of 1:300. Staining visualization was achieved by reaction with the Vectastain ABC kit (PK-4000, Vector Laboratories) and 3,3′-diaminobenzidine tetrahydrochloride (DAB, D5905, Sigma-Aldrich). Dehydration of the mounted slides was achieved using Citrisolv Clearing Agent (22-143-975, Decon Labs) and slides were coverslipped using Cytoseal (8310-4, Thermo Fisher Scientific). The number of c-Fos-positive cells in the blades of the DG were counted live on a Leica DM5500 B Upright Microscope at 20× magnification by an experimenter blinded to treatment. Representative images were acquired with the Hamamatsu Nanozoomer 2.0HT at 20×.

### iDISCO

All experimental procedures were performed at Certerra. Mice were anesthetized with ketamine/xylazine 2.5 h after last dose and subsequently perfused transcardially with 0.9% saline followed by 4% formaldehyde. The brains were postfixed then processed with the 3D imaging of solvent-cleared organs (iDISCO) procedure using the c-Fos antibody (2250, Cell Signaling Technology) at a concentration of 1:1000 followed by incubation with appropriate secondary. Cleared samples were imaged in sagittal orientation (right lateral side up) on a light-sheet fluorescence microscope (Ultramicroscope II, LaVision Biotec) equipped with a sCMOS camera (Andor Technology) and a 4×/0.5 objective lens (MVPLAPO 4×) equipped with a 6-mm working distance dipping cap. Version v144 of the Imspector Microscope controller software was used. The samples were scanned with a step size of 3 μm using the continuous light-sheet scanning method with the included contrast blending algorithm for the 640- and 595-nm channels (20 acquisitions per plane), and without horizontal scanning for the 480-nm channel.

### ELISA

For acute plasma concentration experiments, blood was collected from the submandibular vein into tubes containing ethylenediaminetetraacetic acid (EDTA, BM-711, Boston BioProducts). For chronic administration experiments, terminal blood was collected by cardiac puncture in syringes containing EDTA (BM-711, Boston BioProducts). Plasma was isolated by centrifugation at 1000 × *g* for 15 min at 4°C and immediately frozen on dry ice. Hemibrain lysates were homogenized in Tissue Extraction Reagent I (FNN0071, Thermo Fisher Scientific). Tissue was homogenized using a Bead Ruptor (Omni International), homogenates were centrifuged at max speed (∼21,330 × *g*) for 20 min at 4°C, and then supernatants were collected for subsequent analysis of the soluble fraction.

Mouse TIMP2 levels were detected in plasma diluted 1:5000 using a mouse TIMP-2 DuoSet ELISA (DY6304, R&D Systems) and DuoSet Ancillary Reagent Kit 2 (DY008, R&D Systems). Mouse MMP2 levels were detected in plasma diluted 1:100 using a mouse MMP-2 ELISA (RAB0366, Sigma-Aldrich). Human TIMP2 levels in the plasma and brain were detected using a human TIMP-2 DuoSet ELISA (DY971, R&D Systems) and DuoSet Ancillary Reagent Kit 2 (DY008, R&D Systems). For acute low dose and chronic administration experiments (250 μg/kg), plasma was diluted 1:25. For acute high dose experiments (1 mg/kg), hemibrain tissue was diluted 1:3. All samples were run in duplicate and the ELISA plates were read on a BMG LABTECH CLARIOstar plate reader at 450 nm and wavelength correction set at 540 nm.

### qPCR

RNA was isolated from hippocampal brain tissue using the RNeasy Mini kit (74106, QIAGEN) according to the manufacturer’s instructions. Briefly, tissue was homogenized in RLT buffer using a Bead Ruptor, and then RNA was bound to an RNA isolation column, washed, and eluted. Contaminating DNA was removed by DNase digestion and cDNA was generated using the Superscript III First-Strand Synthesis SuperMix kit (11752050, Invitrogen). A master mix for qPCR was made using SYBR Green reagent (43-091-55, Fisher Scientific) or TaqMan multiplex reagent (44-842-63, Fisher Scientific) and the appropriate forward and reverse primers, and the reactions were run in technical triplicates. The reaction was run on a QuantStudio Flex Real-Time PCR System (Applied Biosystems) and analyzed using the std ddCT protocol on the QuantStudio 6 software (Applied Biosystems) by a single experimenter blinded to treatment.

### MMP activity assays

A Eurofins Panlabs Discovery, MMP panel (PP258) was used to analyze protein construct inhibitory activity on the following human MMPs: MMP1, MMP2, MMP3, MMP7, MMP8, MMP9, MMP10, MMP12, MMP13, MMP14, MMP15, MMP17, MMP19, MMP20, and MMP24. TIMP2, TIMP-hIgG4, and Ala-TIMP2 were shipped from Alkahest to Taipei, Taiwan on dry ice and assessed at the following concentrations: 4000 nm, 250 nm, and 10 nm.

MMP activity was assessed at Alkahest using activity kits from AnasSpec as follows. The ability of the protein constructs to inhibit MMP2 was analyzed using the SensoLyte 520 MMP-2 Assay kit (AS-71151, AnaSpec) and recombinant human MMP-2 pro-enzyme (Hu-pro-MMP2.0, 902-MP-010, R&D Systems; Hu-pro-MMP2.2, AS-72005, AnaSpec). The MMP-2 pro-enzyme was activated using 4-aminophenylmercuric acetate (APMA) according to manufacturer’s instructions. TIMP2 and TIMP2-hIgG4 were tested at the following concentrations: 500 nm, 166.7 nm, 55.6 nm, 18.5 nm, 6.2 nm, 2.1 nm, 0.69 nm, and 0.23 nm. Ala-TIMP2 was tested at the following concentrations: 4000 nm, 1500 nm, 1000 nm, 500 nm, 150 nm, 50 nm, and 25 nm. The fluorescent intensity of the plate was read on a BMG LABTECH CLARIOstar plate reader at Ex/Ex = 490/520 nm after 15 min of initiating the enzymatic reaction.

The ability of the protein constructs to inhibit MMP3 was analyzed using the SensoLyte 520 MMP-3 Assay kit (AS-71152, AnaSpec) and recombinant human MMP-3 catalytic domain (Hu-CD-MMP3, AS-72006, AnaSpec). TIMP2, Ala-TIMP2, and TIMP2-hIgG4 were tested at the following concentrations: 4000 nm, 1500 nm, 1000 nm, 500 nm, 250 nm, 50 nm, 25 nm, 10 nm, and 2.5 nm. The fluorescent intensity of the plate was read on a BMG LABTECH CLARIOstar plate reader at Ex/Ex = 490/520 nm after 15 min of initiating the enzymatic reaction.

The ability of the protein constructs to inhibit MMP9 was analyzed using the SensoLyte 520 MMP-9 Assay kit (AS-71155, AnaSpec) and recombinant human MMP-9 catalytic domain (Hu-CD-MMP9, AS-55576-1, AnaSpec). TIMP2, Ala-TIMP2, and TIMP2-hIgG4 were tested at the following concentrations: 4000 nm, 1500 nm, 1000 nm, 500 nm, 250 nm, 50 nm, 25 nm, 10 nm, and 2.5 nm. The fluorescent intensity of the plate was read on a BMG LABTECH CLARIOstar plate reader at Ex/Ex = 490/520 nm after 75 min of initiating the enzymatic reaction.

Substrate only and substrate plus MMP controls were used to calculate the percent inhibition, as follows:

$$\text{\% MMP inhibition}=100-\frac{\left(\gamma 490,sample-\gamma 520,sample)-(\gamma 490,substrate-\gamma 520,substrate\right)}{\left(\gamma 490,MMP-\gamma 520,MMP)-(\gamma 490,substrate-\gamma 520,substrate\right)}*100.$$

The IC_50_ values were calculated using nonlinear regression of the log of the protein construct concentration versus the percent MMP inhibition with variable slope and four parameters.

### MMP binding assays

#### MMP proteins

Recombinant MMPs were derived from mouse and human. Mouse (Ms-) MMP2 (Ms-pro-MMP2, 554404, BioLegend), MMP3 (Ms-pro-MMP3, 552704, BioLegend), and MMP9 (Ms-pro-MMP9, 755204, BioLegend) were all the zymogen (pro-) forms of the enzymes at ∼73, 54, and 80 kDa, respectively. Human (Hu-) MMP2 (Hu-pro-MMP2.1, 554304, BioLegend; Hu-pro-MMP2.2, AS-72005, AnaSpec), MMP3 (Hu-pro-MMP3, 594704, BioLegend), and MMP9 (Hu-pro-MMP9, 550504, BioLegend) included the pro- forms of the enzymes, as well as the catalytic domains (CD-) of MMP3 (Hu-CD-MMP3, AS-72006, AnaSpec) and MMP9 (Hu-CD-MMP9, AS-55576-1, AnaSpec). The catalytic only domains of MMP3 and MMP9 had reduced molecular weights (MWs) from ∼54 to ∼28 kDa and ∼80 to ∼40 kDa, respectively. Bio-layer interferometry (BLI) studies assessed all the aforementioned MMP versions, while surface plasmon resonance (SPR) studies excluded those derived from mouse because of experimental constraints.

#### Octet

Analysis via bio-layer interferometry (BLI) with the Octet Red96e (Pall ForteBio, currently Sartorius, Göttingen, Germany) was used to obtain kinetics and affinity data and further used in binning analysis. TIMP2 and TIMP2-hIgG4 proteins were biotinylated by molecular weight (MW) with a 1:1 molar ratio of biotin to protein using the EZ-Link NHS-PEG4-Biotin, No-Weigh Format Biotinlyation kit (A39259, Thermo Fisher Scientific). Biotinylated proteins (ligand) were prepared at 45 nm concentrations in 1× PBS, 3% BSA (A7888-100g, Sigma-Aldrich), 0.02% Tween 20 (P-7949, Sigma-Aldrich; running buffer) and loaded onto Streptavidin Capture tips (18-0009, Sartorius). MMP proteins (analyte) were prepared at 22.5 nm in running buffer. Ligand was associated with analyte for 180 s. Complex formation was then allowed to dissociate into running buffer. Changes in nm shift because of binding were recorded for subsequent analysis.

#### Biacore

The Biacore T200 (Cytiva) was used for surface plasmon resonance (SPR) assays. Stock solutions of purified TIMP2, Ala-TIMP2, and TIMP2-hIgG4 were prepared in 10 mm sodium acetate buffer, pH 5.0 (BR100351, Cytiva), at concentrations of 100 nm and individually immobilized. TIMP2 and Ala-TIMP2 were targeted for the same immobilization level to a Series S CM5 sensor chip (29149603, Cytiva) using the amine coupling reaction as described by the manufacturer (BR00050, Cytiva). The reaction used EDC and NHS chemistries and the remaining binding sites were blocked with 1 M ethanolamine, pH 8.5. TIMP2-hIgG4 was targeted for the same immobilization level as TIMP2 and Ala-TIMP2 but multiplied by the MW ratio of TIMP2-hIgG4 to TIMP2. A control flow cell was also made for each TIMP variant. Single cycle kinetics assays were performed as described by the manufacturer at 25°C in HBS-P+ buffer [10 mm HEPES pH 7.4, 150 mm NaCl, 0.05% (v/v) Surfactant P20, BR100671, Cytiva] at a flow rate of 30 μl/min for recombinant human MMP analytes from BioLegend (Hu-pro-MMP2.1, 554304; Hu-pro-MMP3, 594704; Hu-pro-MMP9, 550504) at five concentrations (200 nm, 100 nm, 50 nm, 25 nm, and 12.5 nm in HBS-P+). High-performance binding assays were performed with the same HBS-P+ buffer and 5 μl/min for recombinant human MMP analytes from AnaSpec (Hu-pro-MMP2.2, AS-72005; Hu-CD-MMP3, AS-72006; Hu-CD-MMP9, AS-55576-1) at concentrations of 20 nm in HBS-P+. Injection times for the latter were 2 min followed by 10 min of dissociation. Regeneration of MMP2 from BioLegend and AnaSpec were performed after each binding cycle using a 4.5-min pulse of 10 mm glycine-HCl, pH 2.5 (BR100356, Cytiva), with a 30 s waiting period. For all other ligands, regeneration was performed using 30 s of 10 mm glycine-HCl, pH 2.5 (BR100356, Cytiva), with a 30-s waiting period. Binding observed in the SPR experiments was assessed by both binding level (the amount of analyte bound immediately before halting analyte flow over the CM5 chip, an average of a 5-s window) and binding stability (the amount of analyte remaining bound after washing with running buffer for 10 s, an average of a 5-s window).

### Experimental design and statistical analysis

All data were analyzed using GraphPad Prism 8 (GraphPad Software). Sample sizes used were comparable to those employed in the field and all experimental n values reflect biological replicates of individual mice unless otherwise stated. For *n* > 10 with normally distributed data, parametric tests were used, otherwise nonparametric tests were used. If technical replicates were used, it is stated explicitly within the methods section. Technical replicates reflect sample replicates from the same mouse, such as region of interest (ROI). Statistical significance was defined as *p* < 0.05.

For measurements of endogenous mouse protein levels of TIMP2 in plasma, the data were analyzed using a one-way ANOVA followed by Dunnett’s multiple comparisons test. For measurements of endogenous mouse protein levels of MMP2 in plasma, the data were analyzed using a Kruskal–Wallis test followed by Dunn’s multiple comparisons test.

For nesting analysis, animals were divided into two groups of either scores of 5 (complete, dome-like nest) or scores below 5 (flat nest). Percent nest scores of 5 or scores below 5 for each treatment group were calculated, and χ^2^ tests were used to test statistical significance between two groups. Mouse body weight was analyzed by a mixed-effects model with main effects of treatment and time. Survival was analyzed by a log-rank (Mantel–Cox) test.

For Y-maze, percent novel entries for each group were compared against 50% chance using one-sample Wilcoxon signed-rank tests. Total distance traveled and average velocity in Y-maze were analyzed using Kruskal–Wallis tests followed by Dunn’s multiple comparisons tests. Mice that did not move during the Y-maze task were excluded from analysis.

The total number of c-Fos-positive and DCX-positive cells per dentate gyrus were estimated by counting the number of positive cells from six tissue sections and multiplying the sum of the number counted per section by 12, as an estimate for the total hippocampal volume. Mice with less than six sections were excluded from the analysis. Both c-Fos and DCX were analyzed using Kruskal–Wallis tests followed by Dunn’s multiple comparisons test. The thresholded percent area of EGR1, CD68, and Iba1 were measured from 5–6 hippocampi per mouse using Image-Pro 9.2 software (Media Cybernetics). Individual sections within a mouse were excluded if they were an outlier based on a regression and outlier removal test (ROUT; Q = 1%). Mice with less than five quantifiable sections were excluded from the analysis. EGR1, CD68, and Iba1 were analyzed using nested one-way ANOVAs followed by Dunnett’s multiple comparisons test.

Synapsin/PSD-95 excitatory synapses were analyzed from five to six ROIs in the stratum radiatum of the CA1 hippocampal region and 6–10 ROIs from the hilus of the DG hippocampal region. Homer1/PSD-95 excitatory synapses were analyzed from six to eight ROIs in the CA1 hippocampal region and six to eight ROIs from the DG hippocampal region. Gephyrin/PSD-95 inhibitory synapses were analyzed from six ROIs in the CA1 hippocampal region and five to six ROIs from the DG hippocampal region. For all synaptic data, outliers were excluded following a ROUT test (Q = 1%) and then analyzed by Kruskal–Wallis tests followed by Dunn’s multiple comparisons test.

For iDISCO c-Fos analysis, statistical comparisons between different groups were run based on ROIs. The cell counts at a given ROI, Y, were assumed to follow a negative binomial distribution whose mean is linearly related to one or more experimental conditions, X: E[Y] = α + βX. For example, when testing an experimental group versus a control group, the X is a single column showing the categorical classification of mouse sample to group ID, i.e., 0 for the control group and 1 for the experimental group (O’Hara and Kotze, 2010; Venables and Ripley, 2002). The maximum likelihood coefficients α and β were found through iterative reweighted least squares, obtaining estimates for sample standard deviations in the process, from which the significance of the β coefficient was obtained. A significant β means the group status is related to the cell count intensity at the specified location. To account for multiple comparisons across all ROI locations, the p-values and reported false discovery rates (FDRs) were thresholded with the Benjamini-Hochberg procedure using a false discovery rate (FDR) correction of -q < 0.05 (Benjamini and Hochberg, 1995). In contrast to correcting for type I error rates, this method controls the number of false positives among the tests that have been deemed significant. Finally, the data for the whole brain c-Fos counts was analyzed using a Mann–Whitney *U* test.

For gene expression, data were normalized to vehicle control and then analyzed using either Kruskal–Wallis tests followed by Dunn’s multiple comparisons test or using one-way ANOVAs followed by Dunnett’s multiple comparisons test. Samples were excluded from the final analysis if the standard deviation between triplicates was >1 or if the average of the triplicates was an outlier based on a ROUT outlier test (Q = 1%).

For the high dose TIMP2 brain penetrance experiment, human TIMP2 detected in the hemibrain lysate of the TIMP2 and TIMP2-hIgG4 treatment groups was analyzed by two-way ANOVA with main effects of treatment and time followed by Sidak’s multiple comparisons test.

## Results

### Fusion to hIgG4 extended the half-life of TIMP2 while retaining the beneficial cognitive effects in aged C57BL/6J mice

**Figure 1.**
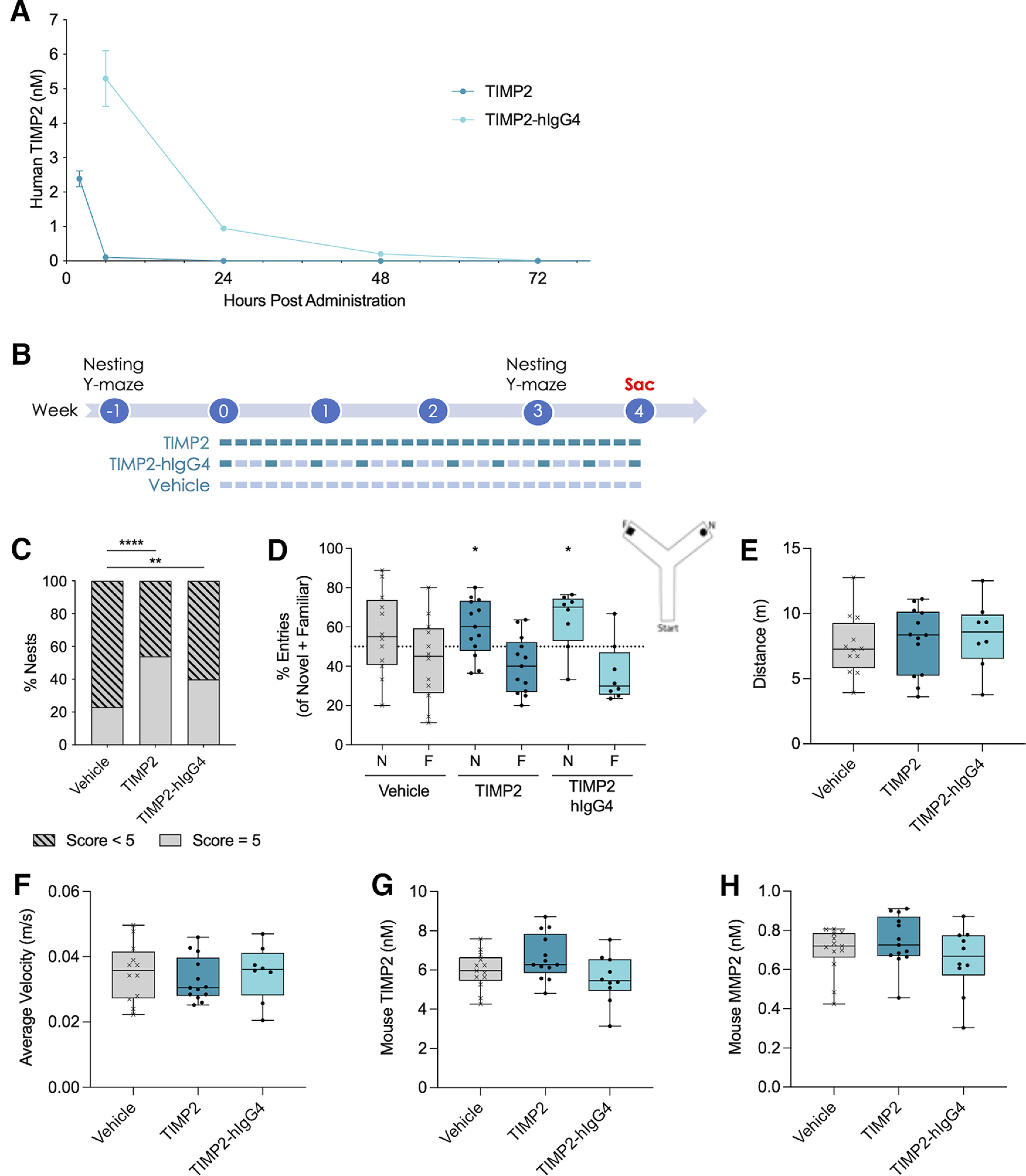
Treatment with TIMP2 and the TIMP2-hIgG4 fusion protein improved nesting and memory in the hippocampal-dependent memory task Y-maze. ***A***, Human TIMP2 in mouse plasma after a single administration of protein (250 μg/kg). *n* = 2–3 mice per time point. ***B***, Timeline for chronic administration experiments. Mice were homogenized based on pretreatment nesting and Y-maze performance then dosed for four weeks. TIMP2 protein and vehicle (DPBS) were administered daily while TIMP2-hIgG4 was administered every third day with vehicle on the off days. Posttreatment nesting and Y-maze assessment occurred during week 3. Sac, sacrifice. ***C***, Percent nest scores of 5 (dome-like, complete nests) or <5 (flat nests) following three weeks of treatment. *n* = 10–13 mice per group. χ^2^ tests: Vehicle versus TIMP2 χ^2^(1, *N* = 26) = 20.29, ****p < 0.0001; Vehicle versus TIMP2-hIgG4 χ^2^(1, *N* = 23) = 6.697, **p = 0.0097. ***D***, Average percent entries into the novel (N) and familiar (F) arms of Y-maze during the testing phase for each treatment group following three weeks of treatment. *n* = 8–13 mice per group. One-sample Wilcoxon signed-rank tests to compare the percent of novel entries for each group against 50% chance: Vehicle *W* = 23.00 *p* = 0.3301; TIMP2 *W* = 52.00, *p = 0.0425; TIMP2-hIgG4 *W* = 24.00, *p = 0.0469. Schematic depicting Y-maze set up. ***E***, Total distance traveled in 5 min during the testing phase of Y-maze. *n* = 8–13 mice per group. Kruskal–Wallis test *H*(2) = 0.8808, *p* = 0.6438. ***F***, Average velocity over 5 min during the testing phase of Y-maze. *n* = 8–13 mice per group. Kruskal–Wallis test *H*(2) = 0.4906, *p* = 0.7825. ***G***, Endogenous mouse TIMP2 protein in plasma after four weeks of chronic administration of human TIMP2 protein. *n* = 10–13 per group. One-way ANOVA *F*_(2,33)_ = 2.997, *p* = 0.0637. ***H***, Endogenous mouse MMP2 protein in plasma after four weeks of chronic administration of human TIMP2 protein. *n* = 10–13 per group. Kruskal–Wallis test *H*(2) = 2.018, *p* = 0.3646. Data for A are shown as mean ± SEM, while box plots include horizontal lines representing the 25th, 50th (median), and 75th percentiles.

Peripherally administered TIMP2 has a short half-life of 4.33 h in blood (Castellano et al., 2017), making the translation of recombinant TIMP2 therapeutics into humans challenging. To extend the half-life of TIMP2 in aged mice, a fusion protein construct to human IgG4Fc (TIMP2-hIgG4) was generated. Following a single IP injection of 250 μg/kg, TIMP2-hIgG4 protein levels were ∼50-fold higher at 6 h postinjection relative to TIMP2 alone and reached undetectable levels by 72 h (Fig. 1*A*).

To determine whether the hIgG4 fusion to TIMP2 impacted the beneficial effects on cognition in aged male C57BL/6J mice, TIMP2 or TIMP2-hIgG4 was chronically administered to 23-month-old mice for one month. Based on the PK findings (Fig. 1*A*), TIMP2 protein was dosed daily while TIMP2-hIgG4 protein was dosed every third day to achieve comparable terminal plasma levels (Fig. 1*B*). First, to determine the effects of treatment on natural behaviors and health, animals were scored on their ability to build nests in a range of 1–5. Animals were divided into two groups of either those that made complete, dome-like nests (scores of 5) or flat nests (scores 1–4). Both TIMP2 (χ^2^(1, *N* = 26) = 20.29, *p* < 0.0001, χ^2^ test) and TIMP2-hIgG4 (χ^2^(1, *N* = 23) = 6.697, *p* = 0.0097, χ^2^ test) treatment significantly improved nesting over vehicle-injected mice (Fig. 1*C*). Next, to determine whether the TIMP2-hIgG4 construct retained its beneficial effect on cognition, mice were tested in the hippocampal-dependent spatial learning and memory task Y-maze. Animals treated with TIMP2 (*W* = 52.00, *p* = 0.0425, one-sample Wilcoxon signed-rank test) and TIMP2-hIgG4 (*W* = 24.00, *p* = 0.0469, one-sample Wilcoxon signed-rank test) but not vehicle (*W* = 23.00, *p* = 0.3301, one-sample Wilcoxon signed-rank test) had significantly more percent entries into the novel arm compared with 50% chance, indicating that vehicle-treated mice were not able to remember the novel arm while TIMP2 and TIMP2-hIgG4 treated animals did (Fig. 1*D*). There were no differences in distance traveled (*H*(2) = 0.8808, *p* = 0.6438, Kruskal–Wallis test; Fig. 1*E*) or average velocity (*H*(2) = 0.4906, *p* = 0.7825, Kruskal–Wallis test; Fig. 1*F*) between treatment groups, suggesting no effect on anxiety or motor functions.

With this chronic dosing paradigm, neither endogenous TIMP2 (*F*_(2,33)_ = 2.997, *p* = 0.0637, one-way ANOVA; Fig. 1*G*) nor MMP2 (*H*(2) = 2.018, *p* = 0.3646, Kruskal–Wallis test; Fig. 1*H*) protein concentration in plasma were altered, suggesting that any effects of treatment were because of the administered human TIMP2 protein and not because of changes in endogenous levels of TIMP2 or MMP2. Additionally, there were no differences between treatment groups in body weight over the course of treatment (Treatment main effect *F*_(2,45)_ = 0.2142, *p* = 0.8080; Time main effect *F*_(4.147,179.6)_ = 87.90, *p* < 0.0001; Treatment × Time interaction *F*_(38,823)_ = 0.6908, *p* = 0.9219; mixed-effects model) or survival [χ^2^(2) = 0.6783, *p* = 0.7124, log-rank (Mantel–Cox) test], indicating there were no overt detrimental health effects with this dosing paradigm.

These data suggest that peripherally administered TIMP2 and TIMP2-hIgG4 treatment resulted in behavioral improvements in aged C57BL/6J mice, including in natural behaviors such as nesting and in cognitive and memory related behaviors. This corroborates findings in previous publications on the impact of TIMP2 on cognition (Castellano et al., 2017) and further extends these findings to TIMP2-hIgG4 with an extended half-life.

### TIMP2 treatment increased *cfos* gene expression without altering neurogenesis markers or immediate early gene products

**Figure 2.**
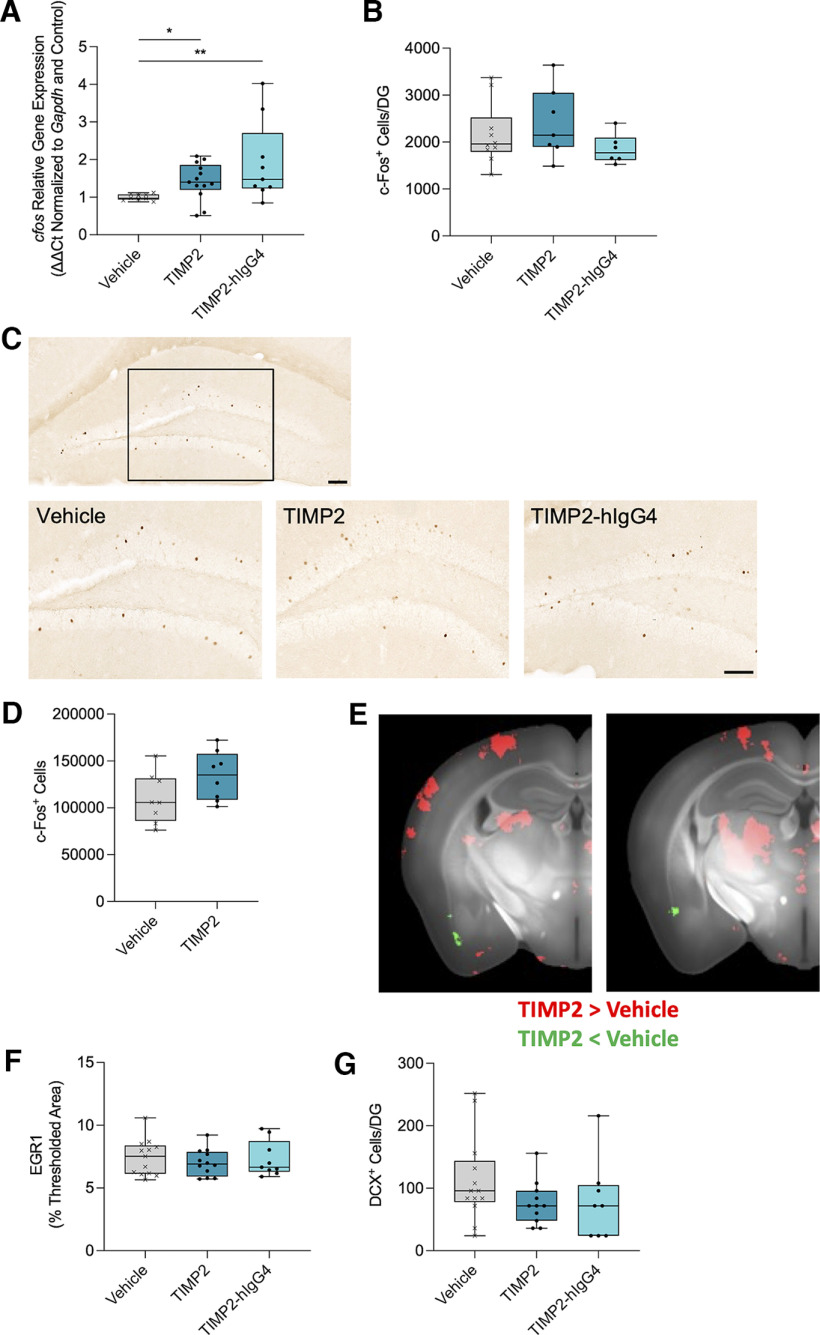
TIMP2 treatment increased *cfos* gene expression without altering neurogenesis markers or immediate early gene products in the hippocampus. ***A***, Average hippocampal *cfos* gene expression relative to *Gapdh* measured by Taqman qPCR for each treatment group. *n* = 9–13 mice per group. Kruskal–Wallis test *H*(2) = 9.773, *p* = 0.0075, followed by Dunn’s multiple comparisons test: Vehicle versus TIMP2 *p = 0.0173, Vehicle versus TIMP2-hIgG4 **p = 0.0087. ***B***, Number of c-Fos-positive (c-Fos+) cells per dentate gyrus (DG) for each treatment group. *n* = 6–10 mice per group. Kruskal–Wallis test *H*(2) = 2.129, *p* = 0.3450. ***C***, Representative images of c-Fos staining in the DG of mice for each treatment group. Scale bar: 100 μm. ***D***, Quantification of c-Fos-positive cells across the entire brain measured following iDISCO procedure. *n* = 8 mice per group. Mann–Whitney *U* test, *U*(n_1_ = n_2_ = 8) = 16, *p* = 0.1049. ***E***, Representative images of c-Fos in cleared brain tissue using iDISCO. Images represent the average of the difference between vehicle and TIMP2 treated mice. Red color represents increased c-Fos in TIMP2 treatment relative to Vehicle and green color represents decreased c-Fos in TIMP2 treatment relative to Vehicle. ***F***, Average thresholded percent area of immediate early gene product early growth response 1 (EGR1) in the hippocampus for each treatment group. *n* = 9–13 mice per group. Nested one-way ANOVA *F*_(2,31)_ = 0.3232, *p* = 0.7263. ***G***, Number of doublecortin-positive (DCX+) cells per dentate gyrus (DG) as a marker of newborn neurons for each treatment group. *n* = 8–13 mice per group. Kruskal–Wallis test *H*(2) = 3.126, *p* = 0.2095. Box plots include horizontal lines representing the 25th, 50th (median), and 75th percentiles.

The mechanism for TIMP2 improvement in cognition has been hypothesized to be because of an increase in neuronal activity via c-Fos without changing neurogenesis (Castellano et al., 2017). A modest increase in *cfos* gene expression in bulk hippocampal tissue (*H*(2) = 9.773, *p* = 0.0075, Kruskal–Wallis test) was identified with both TIMP2 (*p* = 0.0173, Dunn’s *post hoc* test) and TIMP2-hIgG4 administration (*p* = 0.0087, Dunn’s *post hoc* test; Fig. 2*A*; Tables 4, 5). However, there were no differences in the number of c-Fos-positive cells in the blades of the DG of the hippocampus as measured by histology (*H*(2) = 2.129, *p* = 0.3450, Kruskal–Wallis test; Fig. 2*B*,*C*). To further explore the effect of TIMP2 treatment on c-Fos, a separate cohort of mice were treated with 50 μg/kg TIMP2 for 7 d, then brains were processed using an iDISCO procedure, which allows for 3D imaging of immunolabeled markers across the entire brain. TIMP2 treatment showed a small, nonsignificant increase (*U*(n_1_ = n_2_ = 8) = 16, *p* = 0.1049, Mann–Whitney *U* test) in the number of c-Fos-positive cells in the total brain volume compared with saline controls (Fig. 2*D*,*E*; Movie 1).

**Table 4:** Hippocampal gene expression following treatment with TIMP2 and TIMP2-hIgG4

Modality	Gene	Vehicle	TIMP2	TIMP2-hIgG4
Neuronal	*Dcx*	1 ± 0.038	0.924 ± 0.059	1.169 ± 0.077
	*Tubb3*	1 ± 0.030	1.032 ± 0.019	1.087 ± 0.031
	*Syn1*	1 ± 0.041	0.865 ± 0.023	1.060 ± 0.048
	*Dlg4*	1 ± 0.038	0.879 ± 0.032	0.953 ± 0.048
	*Gria1*	1 ± 0.051	1.050 ± 0.085	0.870 ± 0.087
	*Grin2a*	1 ± 0.036	0.977 ± 0.055	0.890 ± 0.039
	*Slc2a1*	1 ± 0.035	0.906 ± 0.026	1.056 ± 0.041
	*Gad1*	1 ± 0.033	1.059 ± 0.039	0.0919 ± 0.071
Immediate early genes	*Cfos*	1 ± 0.028	1.421 ± 0.136	1.923 ± 0.357
	*Creb1*	1 ± 0.027	1.052 ± 0.024	0.940 ± 0.057
	*Egr1*	1 ± 0.073	1.089 ± 0.068	1.387 ± 0.192
Microglia	*Cd68*	1 ± 0.085	1.096 ± 0.080	0.955 ± 0.053
	*Iba1*	1 ± 0.061	0.950 ± 0.060	0.960 ± 0.079
	*Cd11b*	1 ± 0.057	1.176 ± 0.119	0.903 ± 0.110
Inflammatory	*Il1a*	1 ± 0.089	1.086 ± 0.071	0.938 ± 0.105
	*Il1b*	1 ± 0.239	0.884 ± 0.138	0.775 ± 0.184
	*Il6*	1 ± 0.105	0.906 ± 0.069	0.913 ± 0.085
	*Ccl11*	1 ± 0.077	1.174 ± 0.072	0.987 ± 0.080
	*Nfkb*	1 ± 0.039	0.960 ± 0.053	0.879 ± 0.060
	*Tnfa*	1 ± 0.119	0.874 ± 0.141	0.878 ± 0.108
Astrocytes	*Gfap*	1 ± 0.071	1.025 ± 0.071	0.879 ± 0.078
	*Aqp4*	1 ± 0.042	1.067 ± 0.063	0.850 ± 0.079
	*Ggta1*	1 ± 0.066	1.031 ± 0.036	0.809 ± 0.095

Average gene expression relative to *Gapdh* measured by Taqman or SYBR qPCR from bulk hippocampal tissue for each treatment group. *n* = 9–13 mice per group. All data are shown as mean ± SEM.

**Table 5:** Few significant changes in hippocampal gene expression following treatment with TIMP2 or TIMP2-hIgG4

Modality	Gene	TIMP2	TIMP2- hIgG4	Kruskal–Wallis test statistics
Neuronal	*Dcx*	0.6790	0.4208	*H*(2) = 4.646, *p* = 0.0980
	*Tubb3*	0.8189	0.1729	*H*(2) = 2.939, *p* = 0.2300
	*Syn1*	*0.0294	0.9810	*H*(2) = 10.31, *p* = 0.0058
	*Dlg4*	0.0616	0.6662	*H*(2) = 4.667, *p* = 0.0970
	*Gria1*	0.4569	0.8582	*H*(2) = 3.828, *p* = 0.1475
	*Grin2a*	>0.9999	0.1800	*H*(2) = 3.138, *p* = 0.2082
	*Slc2a1*	0.1370	0.9185	*H*(2) = 6.614, *p* = 0.0366
	*Gad1*	0.3957	>0.9999	*H*(2) = 3.438, *p* = 0.1793
Immediate early genes	*Cfos*	*0.0173	**0.0087	*H*(2) = 9.773, *p* = 0.0075
	*Creb1*	0.2678	>0.9999	*H*(2) = 3.817, *p* = 0.1483
	*Egr1*	0.7935	0.1351	*H*(2) = 3.342, *p* = 0.1880
Microglia	*Cd68*	0.6144	>0.9999	*H*(2) = 1.509, *p* = 0.4702
	*Iba1*	>0.9999	>0.9999	*H*(2) = 0.2837, *p* = 0.8678
	*Cd11b*	0.5904	0.9339	*H*(2) = 3.016, *p* = 0.2213
Inflammatory	*Il1a*	0.7900	>0.9999	*H*(2) = 2.050, *p* = 0.3589
	*Il1b*	>0.9999	0.9233	*H*(2) = 0.9005, *p* = 0.6375
	*Il6*	0.5032	>0.9999	*H*(2) = 1.516, *p* = 0.4686
	*Ccl11*	0.1789	>0.9999	*H*(2) = 3.675, *p* = 0.1592
	*Nfkb*	0.9367	0.1776	*H*(2) = 2.903, *p* = 0.2342
	*Tnfa*	0.3888	>0.9999	*H*(2) = 1.687, *p* = 0.4301
Astrocytes	*Gfap*	>0.9999	>0.9999	*H*(2) = 0.7345, *p* = 0.6926
	*Aqp4*	0.2516	0.3938	*H*(2) = 7.558, *p* = 0.0228
	*Ggta1*	>0.9999	0.1737	*H*(2) = 5.146, *p* = 0.0763

Kruskal–Wallis test statistics and *p*-values of *post hoc* Dunn’s multiple comparisons tests for comparisons between average gene expression relative to *Gapdh* measured by Taqman or SYBR qPCR from bulk hippocampal tissue from Vehicle versus the TIMP2 and TIMP2-hIgG4 treatment groups. *n* = 9–13 mice per group.

To determine whether TIMP2 administration impacted other markers of neuronal activity, protein expression of the immediate early gene product early growth response 1 (EGR1) was assessed in the hippocampus. However, there were no changes with treatment as measured by histology (*F*_(2,31)_ = 0.3232, *p* = 0.7263, nested one-way ANOVA; Fig. 2*F*). As previously reported (Castellano et al., 2017), neurogenesis was also unchanged as measured by doublecortin (DCX) labeling of newborn neurons in the blades of the DG (*H*(2) = 3.126, *p* = 0.2095, Kruskal–Wallis test; Fig. 2*G*). Finally, to understand whether TIMP2 treatment broadly impacted other modalities, bulk hippocampal qPCR was performed to examine multiple genes across neurons, microglia, and astrocytes, as well as genes important in inflammatory signaling and immediate early gene responses (Tables 4, 5). There were no significant changes with TIMP2 and TIMP2-hIgG4 treatment across any of these modalities with the exception of *cfos* (Fig. 2*A*; Tables 4, 5), which had been identified previously (Castellano et al., 2017). Overall, these data support the conclusion that effects of TIMP2 and TIMP2-hIgG4 treatment in the brain were not because of large, widespread impacts across multiple cell types, but rather raise the possibility that they could be specific to neurons and neuronal activity.

### TIMP2 treatment increased excitatory synapses but not inhibitory synapses, potentially because of a direct mechanism of action within the brain parenchyma

**Figure 3.**
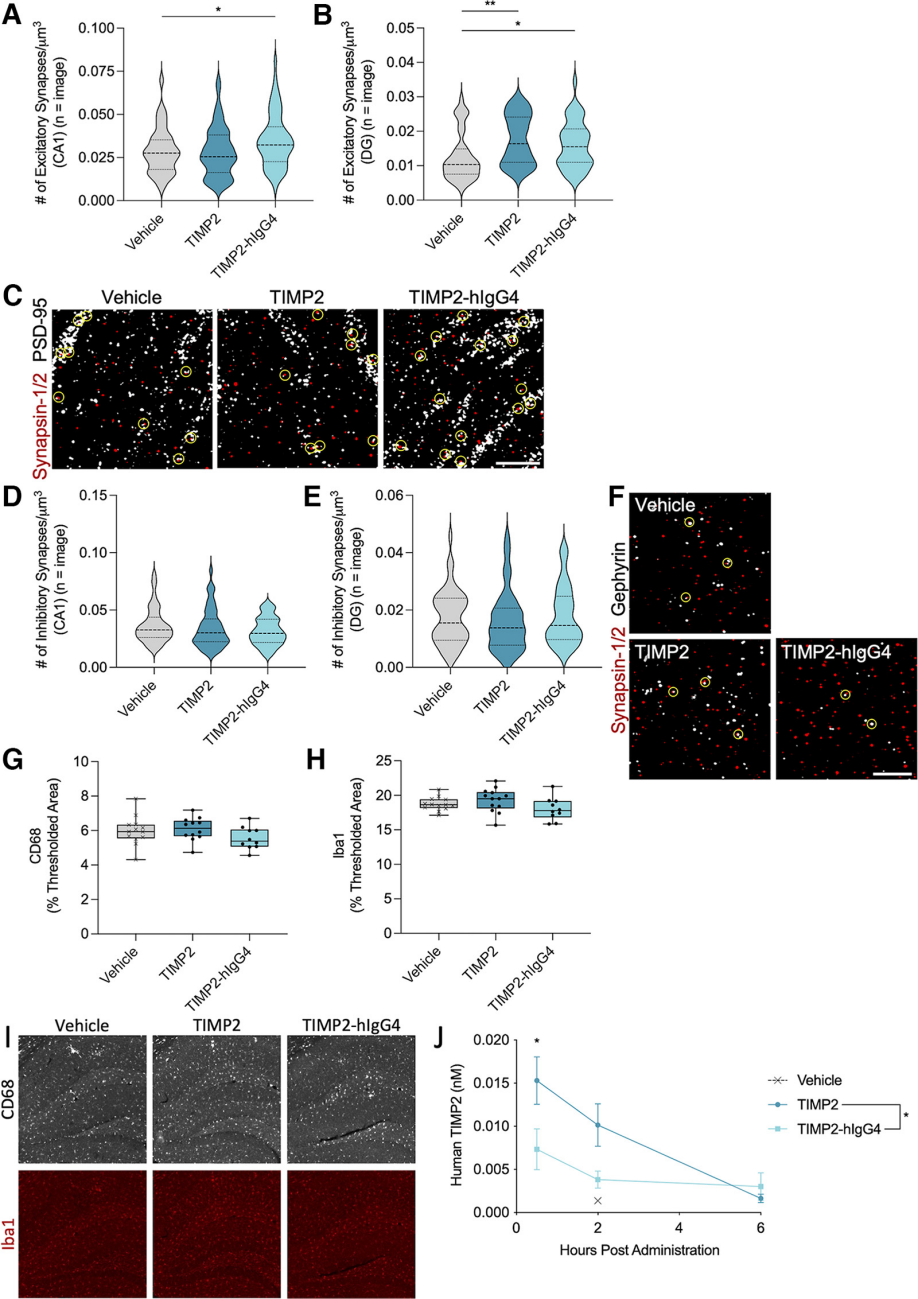
TIMP2 treatment increased excitatory synapses but not inhibitory synapses in the hippocampus, potentially because of a direct mechanism of action within the brain parenchyma. ***A***, Number of juxtaposed presynaptic Synapsin-1/2 and postsynaptic PSD-95 puncta per μm^3^ in the stratum radiatum of the CA1 region of the hippocampus as a readout for excitatory synapse density for each treatment group. *n* = 60–78 images from 10–13 mice per group. Kruskal–Wallis test *H*(2) = 8.004, *p* = 0.0183, followed by Dunn’s multiple comparisons test: Vehicle versus TIMP2 *p* > 0.9999, Vehicle versus TIMP2-hIgG4 *p = 0.0390. ***B***, Number of juxtaposed presynaptic Synapsin-1/2 and postsynaptic PSD-95 puncta per μm^3^ in the hilus of the dentate gyrus (DG) of the hippocampus as a readout for excitatory synapse density for each treatment group. *n* = 42 images from 5 mice per group. Kruskal–Wallis test *H*(2) = 12.15, *p* = 0.0023, followed by Dunn’s multiple comparisons test: Vehicle versus TIMP2 **p = 0.0017, Vehicle versus TIMP2-hIgG4 *p = 0.0231. ***C***, Representative images from the stratum radiatum of the CA1 of a single z-plane of thresholded Synapsin-1/2 (red) and PSD-95 (white) with juxtaposed synapses circled in yellow from each treatment group. Scale bar: 5 μm. ***D***, Number of juxtaposed presynaptic Synapsin-1/2 and postsynaptic Gephyrin puncta per μm^3^ in the stratum radiatum of the CA1 region of the hippocampus as a readout for inhibitory synapse density for each treatment group. *n* = 48 images from 8 mice per group. Kruskal–Wallis test *H*(2) = 2.552, *p* = 0.2791. ***E***, Number of juxtaposed presynaptic Synapsin-1/2 and postsynaptic Gephyrin puncta per μm^3^ in the hilus of the DG of the hippocampus as a readout for inhibitory synapse density for each treatment group. *n* = 47–48 images from 8 mice per group. Kruskal–Wallis test *H*(2) = 1.343, *p* = 0.5110. ***F***, Representative images from the stratum radiatum of the CA1 of a single z-plane of thresholded Synapsin-1/2 (red) and Gephyrin (white) with juxtaposed synapses circled in yellow from each treatment group. Scale bar: 5 μm. ***G***, Average thresholded percent area of CD68-positive microglia in the hippocampus for each treatment group. *n* = 10–13 mice per group. Nested one-way ANOVA *F*_(2,32)_ = 1.589, *p* = 0.2199. ***H***, Average thresholded percent area of Iba1-positive microglia in the hippocampus for each treatment group. *n* = 10–13 mice per group. Nested one-way ANOVA *F*_(2,33)_ = 2.242, *p* = 0.1222. ***I***, Representative images of CD68 (white) and Iba1 (red) microglia from the hippocampus of mice from each treatment group. Scale bar: 100 μm. ***J***, Human TIMP2 in mouse hemibrain after a single high dose administration of protein (1 mg/kg). *n* = 2–3 mice per time point. Two-way ANOVA between TIMP2 and TIMP2-hIgG4 treatment groups: Treatment main effect *F*_(1,12)_ = 7.288, *p = 0.0193; Time main effect *F*_(2,12)_ = 10.59, *p* = 0.0022; Treatment × Time interaction *F*_(2,12)_ = 3.258, *p* = 0.0741. Followed by Sidak’s multiple comparisons test: 0.5 h *p = 0.0407, 2 h *p* = 0.1177, 6 h *p* = 0.9485. Data for ***J*** are shown as mean ± SEM, while violin and box plots include horizontal lines representing the 25th, 50th (median), and 75th percentiles.

To further explore the possible mechanisms underlying behavioral improvements and increased *cfos* gene expression, excitatory synaptic density was assessed by measuring juxtaposed presynaptic Synapsin-1/2 and postsynaptic density protein 95 (PSD-95). Excitatory synapses were significantly increased in the stratum radiatum of the CA1 with TIMP2-hIgG4 treatment (*H*(2) = 8.004, *p* = 0.0183, Kruskal–Wallis test; *p* = 0.0390, Dunn’s *post hoc* test) and increased in the hilus of the DG (*H*(2) = 12.15, *p* = 0.0023, Kruskal–Wallis test) with both TIMP2 (*p* = 0.0017, Dunn’s *post hoc* test) and TIMP2-hIgG4 treatment (*p* = 0.0231, Dunn’s *post hoc* test; Fig. 3*A–C*). Next, to discern whether this effect was specific to excitatory synapses, inhibitory synaptic density was examined via juxtaposed presynaptic Synapsin-1/2 and postsynaptic Gephyrin in the hippocampus. However, inhibitory synapses were unchanged in both the CA1 (*H*(2) = 2.552, *p* = 0.2791, Kruskal–Wallis test; Fig. 3*D*,*F*) and DG (*H*(2) = 1.343, *p* = 0.5110, Kruskal–Wallis test; Fig. 3*E*,*F*). Excitatory synapse density decreases with age and is directly correlated with cognition and memory (Lee et al., 2000; B. Xu et al., 2018), suggesting that this could be one of the underlying mechanisms of improved cognition in aged mice treated with the TIMP2 constructs.

In order to determine whether the administration of a human protein could potentially lead to an inflammatory response in mice as has been reported by others (Jiskoot et al., 2016), microgliosis was measured as percent CD68 (Fig. 3*G*,*I*) and Iba1 (Fig. 3*H*,*I*) in the hippocampus. There were no differences in the TIMP2 groups compared with vehicle-treated mice (CD68: *F*_(2,32)_ = 1.589, *p* = 0.2199, nested one-way ANOVA; Iba1: *F*_(2,33)_ = 2.242, *p* = 0.1222, nested one-way ANOVA). This showed that the administration of human protein did not increase microglia activation of CD68 and Iba1. In addition, microglia are known to undergo multiple changes with age, including proliferation (Long et al., 1998), reactivity (Hefendehl et al., 2014), motility (Damani et al., 2011; Hefendehl et al., 2014), gene expression (Hart et al., 2012; Harry, 2013), and secretion of inflammatory cytokines (Ye and Johnson, 1999; Yu et al., 2002). This heightened neuroinflammatory state can lead to detrimental effects on the CNS and cognitive function (Simen et al., 2011; Rea et al., 2018; Duggan and Parikh, 2021). Since TIMP2 administration did not impact CD68 and Iba1 percent area, this also indicated that TIMP2 treatment is not sufficient to reduce microglia activation and therefore a reduction in microgliosis is not a likely cause of the improved behavior. These results may also suggest that the mechanism of action for the beneficial effects of TIMP2 are not mediated by microglia, but rather could potentially be because of a direct effect on neurons themselves.

To determine whether peripherally administered TIMP2 and TIMP2-hIgG4 could potentially function directly on neurons, mice were administered a single high 1 mg/kg dose of TIMP2 or TIMP2-hIgG4. Both TIMP2 and TIMP2-hIgG4 were detectable in hemibrain lysate after extended perfusion with sterile PBS up to 6 h postadministration (Fig. 3*J*). Thus, it is possible that both TIMP2 and TIMP2-hIGg4 enter the brain parenchyma where they could potentially act directly on neurons.

### Alanine insertion into TIMP2 prevented MMP inhibitory activity at biologically relevant concentrations without affecting MMP binding

The best described function of TIMP2 is the inhibition of MMPs to regulate extracellular matrix degradation (Brew and Nagase, 2010). However, TIMP2 also has reported MMP-independent functions, including the promotion of neuronal differentiation and neurite outgrowth (Pérez-Martínez and Jaworski, 2005), suggesting that the beneficial cognitive effects with TIMP2 treatment may occur through its MMP-independent effects. To test this hypothesis, a TIMP2 construct without MMP inhibitory activity, Ala-TIMP2, was generated by inserting an alanine residue after the signal peptide. This insertion has been reported to provide steric hindrance that prevents inhibition of MMPs by the TIMP2 protein (Wingfield et al., 1999).

To characterize the MMP inhibitory profiles of the TIMP2, TIMP-hIgG4, and Ala-TIMP2 constructs, the constructs were tested in a panel of 15 human MMPs and were found to have distinct MMP inhibitory profiles as measured by percent inhibition (Table 6). Overall, the TIMP2 construct had the highest MMP inhibitory activity, while the Ala-TIMP2 construct had little activity except at the highest 4 μm concentration. The inhibitory activity of each protein construct was further assessed against three MMPs known to interact with TIMP2: MMP2, MMP3, and MMP9. TIMP2 and TIMP2-hIgG4 constructs had similar IC_50_ values across all three MMPs, while the activity of the Ala-TIMP2 construct was greatly reduced (Table 7), indicating that the alanine insertion was sufficient to prevent MMP inhibition by TIMP2 at biologically relevant levels.

**Table 6:** The TIMP2 constructs had distinct MMP inhibitory profiles

% Inhibition	TIMP2			TIMP2-hIgG4			Ala-TIMP2		
	10 nm	250 nm	4000 nm	10 nm	250 nm	4000 nm	10 nm	250 nm	4000 nm
MMP1	1.58	99.30	109.56	−2.28	15.96	107.23	−0.35	0.70	−6.53
MMP2	76.53	99.46	100.98	13.28	99.69	100.49	2.17	7.76	94.59
MMP3	2.80	99.44	99.14	2.06	−0.19	99.43	−0.94	−0.19	95.42
MMP7	−0.67	1.84	100.20	1.34	19.43	100.00	4.19	3.18	89.70
MMP8	9.77	99.64	100.00	6.22	2.49	100.47	−0.71	−4.97	99.53
MMP9	1.21	98.62	100.00	3.28	4.84	99.52	4.15	5.18	98.06
MMP10	−1.83	−1.22	100.19	−0.86	98.28	100.00	1.03	1.20	0.92
MMP12	2.99	97.84	79.74	0.66	4.49	81.70	2.16	−0.33	76.47
MMP13	10.17	81.33	87.89	18.11	1.29	76.65	3.33	5.36	72.03
MMP14	0.89	−2.32	99.40	−3.39	−1.25	99.60	7.62	9.33	50.00
MMP15	91.08	100.20	98.41	26.82	100.00	99.21	0.45	−1.80	98.41
MMP17	50.54	91.30	97.39	27.79	92.99	98.17	−1.82	24.16	74.15
MMP19	1.05	99.34	101.24	−7.32	42.25	100.62	0.63	7.95	22.11
MMP20	0.52	94.50	99.77	3.09	90.03	98.60	−1.20	1.89	56.28
MMP24	41.01	97.36	101.66	30.70	96.88	101.66	11.51	33.09	88.38

Percent inhibition by TIMP2, TIMP-hIgG4, and Ala-TIMP2 on 15 MMPs at three concentrations (4000 nm, 250 nm, and 10 nm).

**Table 7:** The alanine insertion into TIMP2 prevented MMP inhibitory activity at biologically relevant concentrations

IC_50_ (nm)	MMP2	MMP3	MMP9
TIMP2	1.65	7.37	2.00
Ala-TIMP2	2321	1689	127.7
TIMP2-hIgG4	1.82	9.70	1.46e^−5^

The IC_50_ values (nm) of TIMP2, Ala-TIMP2, and TIMP2-hIgG4 inhibitory activity on MMP2, MMP3, and MMP9.

**Figure 4.**
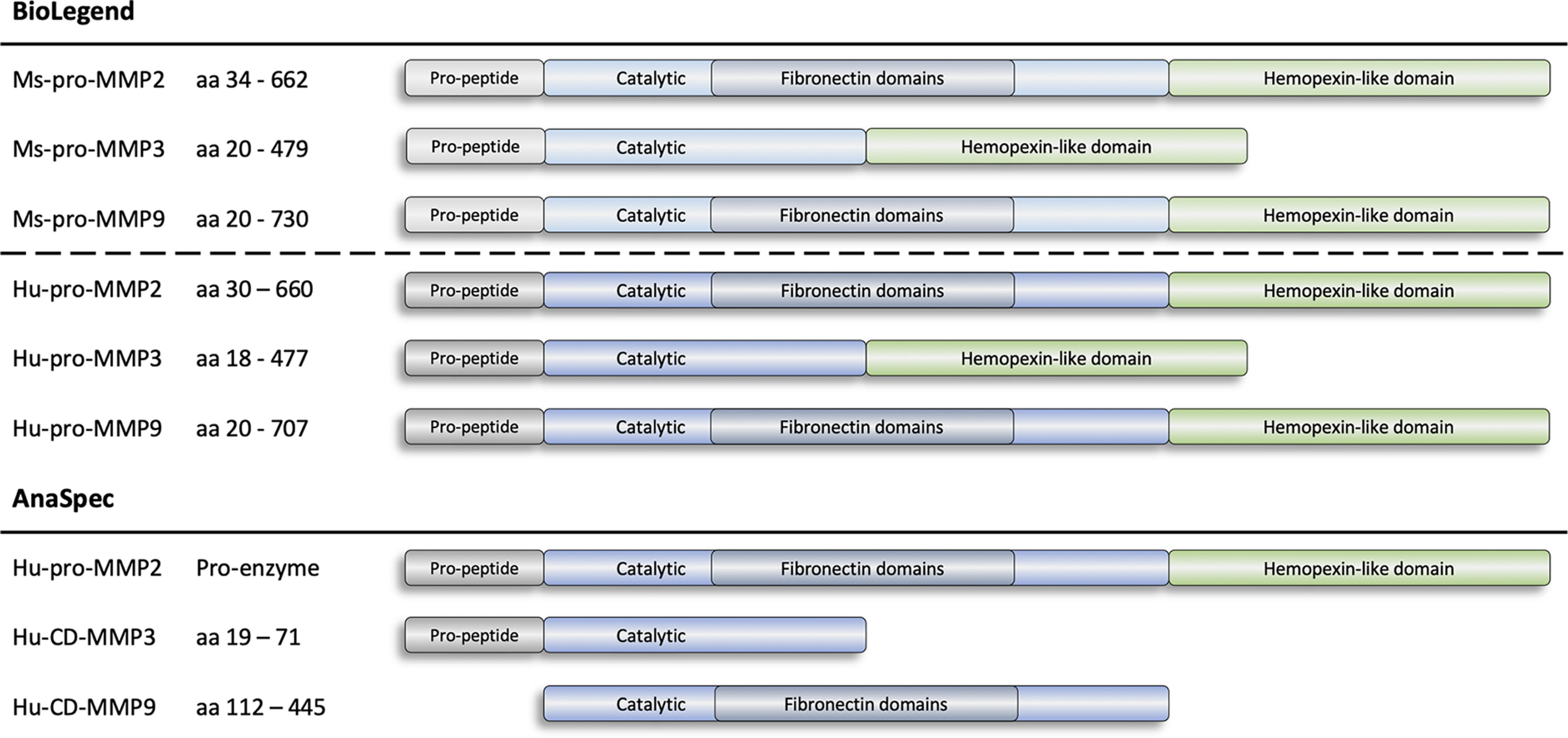
MMP protein constructs. Schematic depicting the nine MMP protein constructs assessed for binding to TIMP2 constructs using bio-layer interferometry (BLI) and surface plasmon resonance (SPR). CD, catalytic domain.

**Figure 5.**
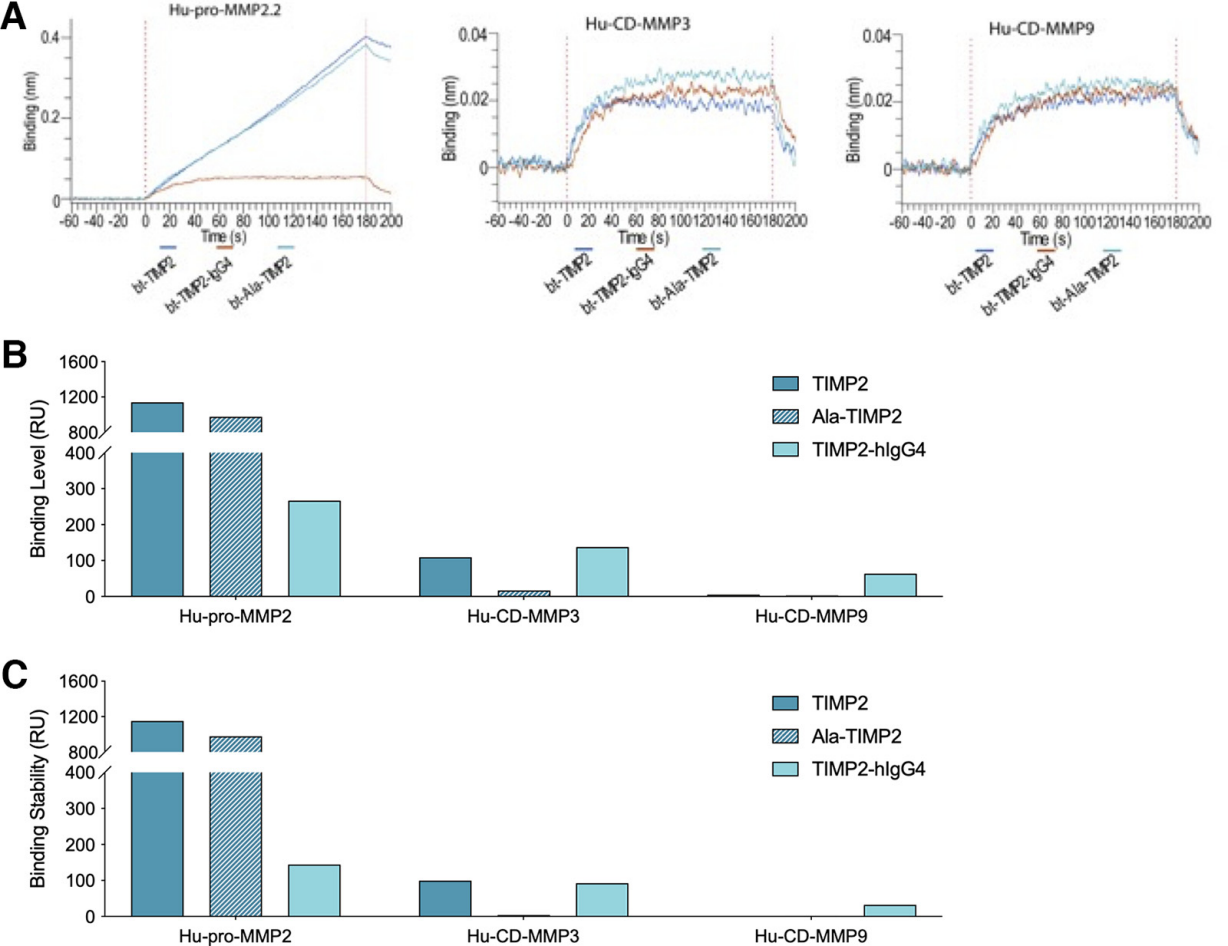
Characterization of TIMP2-MMP binding of the TIMP2 constructs. ***A***, Bio-layer interferometry (BLI) studies were performed on the Octet Red96e. TIMP2, TIMP2-hIgG4, and Ala-TIMP2 were biotinylated with a 1:1 molar ratio of biotin to protein. MMPs were associated at 22.5 nm to captured biotinylated protein on streptavidin tips. Shown here are the binding interactions for Hu-pro-MMP2 (left), Hu-CD-MMP3 (middle), and Hu-CD-MMP9 (right) with the TIMP2 constructs. Significant binding was observed for MMP2. All curves are reference-subtracted. ***B***, Surface plasmon resonance (SPR) bar graph data showing binding level, in response units (RU) of Hu-pro-MMP2, Hu-CD-MMP3, and Hu-CD-MMP9 against the TIMP2 constructs. Values determined from a 5-s window at specified times. Binding level is defined as the RU of a 5-s window average observed immediately before washing the CM5 chip with running buffer, i.e., analyte is still flowing over the CM5ne chip. ***C***, SPR bar graph data showing binding stability, in RU of Hu-pro-MMP2, Hu-CD-MMP3, and Hu-CD-MMP9 against the TIMP2 constructs. Binding stability is defined as RU of a 5-s window average observed after washing the CM5 chip with running buffer for 10 s, i.e., analyte is no longer flowing over the CM5 chip. RU, response unit.

To complement the MMP inhibitory profiles, binding of TIMP2, TIMP2-hIgG4, and Ala-TIMP2 to recombinant MMPs from various sources and forms was tested using bio-layer interferometry (BLI) and surface plasmon resonance (SPR; Fig. 4). BLI results confirmed that TIMP2, Ala-TIMP2, and TIMP2-hIgG4 all have specific binding to Hu-/Ms-pro-MMP2 and had negligible binding to Hu-/Ms-pro-MMP3/9 and Hu-CD-MMP3/9 (Fig. 5*A*; Table 8). This was observed across all sources and forms of MMP enzymes.

**Table 8:** Characterization of TIMP2-MMP binding of the TIMP2 constructs

Ligand	TIMP2		TIMP2-hIgG4		Ala-TIMP2	
Binding method	BLI (nm)	SPR (RU)	BLI (nm)	SPR (RU)	BLI (nm)	SPR (RU)
Ms-pro-MMP2	0.14	N.D.	0.016	N.D.	0.089	N.D.
Ms-pro-MMP3	0.0058	N.D.	0.013	N.D.	0.013	N.D.
Ms-pro-MMP9	0.010	N.D.	0.011	N.D.	0.0044	N.D.
Hu-pro-MMP2.1	0.068	5603.6	0.017	563.87	0.070	4468.99
Hu-pro-MMP3	0.0051	337.9	0.016	249.31	0.014	−85.29
Hu-pro-MMP9	0.013	219.2	0.013	164.32	0.0076	18.63
Hu-pro-MMP2.2	0.38	1140.07	0.079	266.57	0.37	975.75
Hu-CD-MMP3	0.015	109.39	0.035	137.61	0.025	16.64
Hu-CD-MMP9	0.019	5.03	0.025	63.44	0.021	3.10

Binding level was analyzed by bio-layer interferometry (BLI) and surface plasmon resonance (SPR) to determine interaction relationships between the TIMP2 constructs and MMP proteins. Binding interactions were assessed for mouse pro- (zymogen) forms and two human forms [pro-, catalytic domain (CD)] of MMP2, MMP3, and MMP9. BLI was performed on Octet Red96e. SPR was performed on Biacore T200. N.D. = No data collected for that combination. N.B. = No binding observed for that combination. Bindings scale cutoffs determined from largest observed binding signal.

Next, the studies were repeated using SPR with Hu-pro-MMP2 and Hu-CD-MMP3/9, as SPR has the advantage of detecting lower affinity binding interactions, allowing for the possible detection of more subtle binding differences between TIMP2 constructs. Similar to BLI, the SPR results showed that Hu-pro-MMP2 binding level against TIMP2, Ala-TIMP2, and TIMP2-hIgG4 was high relative to their corresponding interactions with Hu-CD-MMP3 and Hu-CD-MMP9 (Fig. 5*B*). Additionally, binding stability was also high for Hu-pro-MMP2 against TIMP2 and Ala-TIMP2 [no reduction in response unit (RU)]; however, there was an ∼56% reduction in RU for Hu-pro-MMP2 against TIMP2-hIgG4, while binding stability for Hu-CD-MMP3 and Hu-CD-MMP9 was minimal (Fig. 5*C*). These results confirm that inserting an alanine residue after the signal peptide of TIMP2 prevents MMP2 inhibition likely via steric hindrance (Wingfield et al., 1999), but does not block binding of the two proteins. Furthermore, TIMP2 had slightly lower inhibitory activity and binding to MMP3 and MMP9. Finally, adding an Fc-tag to TIMP2 (TIMP2-hIgG4) was determined to modestly impact MMP2 binding without substantially impacting the IC_50_ values.

### TIMP2 MMP inhibitory activity was not necessary for hippocampal-dependent memory and synaptic improvements

**Figure 6.**
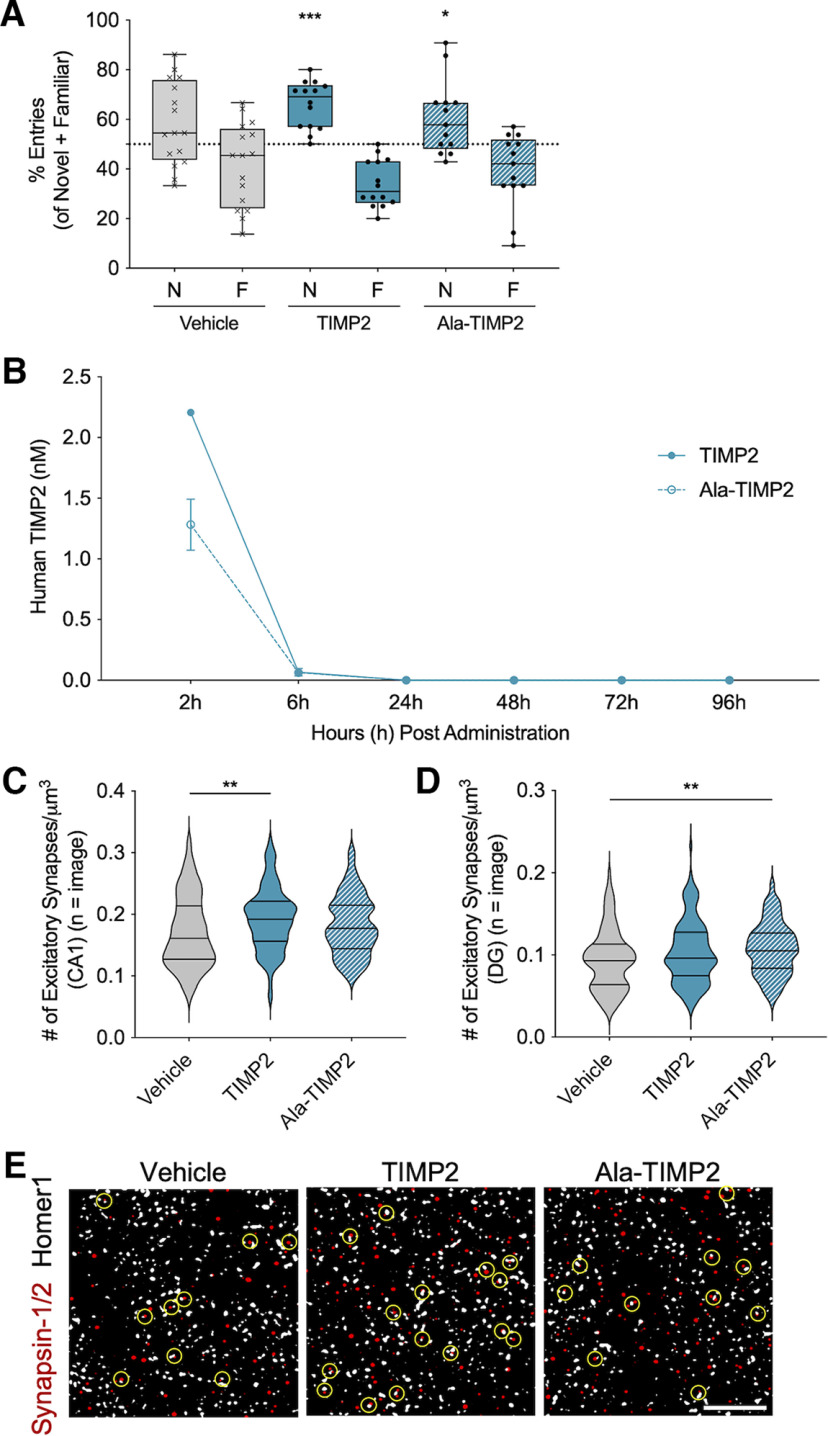
TIMP2 MMP inhibitory activity was not necessary for improvements in hippocampal-dependent memory in Y-maze or hippocampal excitatory synapses. ***A***, Average percent entries into the novel (N) and familiar (F) arms of Y-maze during the testing phase for each treatment group following three weeks of treatment. *n* = 13–16 mice per group. One-sample Wilcoxon signed-rank tests to compare the percent of novel entries for each group against 50% chance: Vehicle *W* = 63.00, *p* = 0.1073; TIMP2 *W* = 91.00, ***p = 0.0002; Ala-TIMP2 *W* = 49.00, *p = 0.0273. ***B***, Human TIMP2 in mouse plasma after a single administration of protein (250 μg/kg). *n* = 1–3 mice per time point. ***C***, Number of juxtaposed presynaptic Synapsin-1/2 and postsynaptic Homer1 puncta per μm^3^ in the stratum radiatum of the CA1 region of the hippocampus as a readout for excitatory synapse density for each treatment group. *n* = 129–139 images from 17–18 mice per group. Kruskal–Wallis test *H*(2) = 11.94, *p* = 0.0026, followed by Dunn’s multiple comparisons test: Vehicle versus TIMP2 **p = 0.0011, Vehicle versus Ala-TIMP2 *p* = 0.1486. ***D***, Number of juxtaposed presynaptic Synapsin-1/2 and postsynaptic Homer1 puncta per μm^3^ in the hilus of the dentate gyrus (DG) of the hippocampus as a readout for excitatory synapse density for each treatment group. *n* = 131–142 images from 17 to 18 mice per group. Kruskal–Wallis test *H*(2) = 10.09, *p* = 0.0064, followed by Dunn’s multiple comparisons test: Vehicle versus TIMP2 *p* = 0.1091, Vehicle versus Ala-TIMP2 **p = 0.0033. ***E***, Representative images from the stratum radiatum of the CA1 of a single z-plane of thresholded Synapsin-1/2 (red) and Homer1 (white) with juxtaposed synapses circled in yellow from each treatment group. Scale bar: 5 μm. Data for ***B*** are shown as mean ± SEM, while box and violin plots include horizontal lines representing the 25th, 50th (median), and 75th percentiles.

To determine whether MMP inhibitory activity is necessary for improvements in cognition *in vivo*, Y-maze was performed in aged mice dosed for one month with TIMP2 or Ala-TIMP2. Mice treated with TIMP2 (*W* = 91.00, *p* = 0.0002, one-sample Wilcoxon signed-rank test) and Ala-TIMP2 (*W* = 49.00, *p* = 0.0273, one-sample Wilcoxon signed-rank test) were able to remember the novel arm as measured by percent entries into the novel arm compared with 50% chance, while vehicle-treated mice (*W* = 63.00, *p* = 0.1073, one-sample Wilcoxon signed-rank test) could not distinguish between the arms (Fig. 6*A*). Furthermore, insertion of the alanine into TIMP2 did not impact the *in vivo* half-life of the protein (Fig. 6*B*), and the concentrations detected in plasma were below the IC_50_ values (Table 7), supporting the conclusion that with the same dosing paradigm, the MMP inhibitory activity of TIMP2 was not essential for the improvement in cognition.

Next, to assess whether MMP inhibitory activity is necessary for improvements in excitatory synaptic density, juxtaposed presynaptic Synapsin-1/2 and postsynaptic Homer1 was measured. Excitatory synapses were significantly increased in the stratum radiatum of the CA1 with TIMP2 treatment (*H*(2) = 11.94, *p* = 0.0026, Kruskal–Wallis test; *p* = 0.0011, Dunn’s *post hoc* test; Fig. 6*C*,*E*) and increased in the hilus of the DG with Ala-TIMP2 treatment (*H*(2) = 10.09, *p* = 0.0064, Kruskal–Wallis test; *p* = 0.0033, Dunn’s *post hoc* test; Fig. 6*D*,*E*). Taken together, these data suggest that MMP inhibition by TIMP2 is not required for the improvements in both excitatory synapses and memory in Y-maze and highlight the likelihood of an MMP-independent mechanism behind these beneficial effects of TIMP2 treatment.

## Discussion

TIMP2 and TIMP2-hIgG4 treatment had beneficial effects on nesting and cognition in Y-maze, similar to previously published reports (Castellano et al., 2017). Additionally, both constructs increased hippocampal *cfos* mRNA, which is consistent with previous findings of increased c-Fos protein expression in the DG following TIMP2 treatment (Castellano et al., 2017). Together these data highlight the reproducibility of the beneficial effects of peripheral TIMP2 administration. However, changes in c-Fos protein expression in the DG were not observed by standard histologic quantification and only subtle changes were measured using the iDISCO technique that allowed for 3D imaging of the whole brain. It is possible that although changes in gene expression were detectable, the time point was not optimized to detect changes by histology. Furthermore, it is possible that different c-Fos antibodies recognize different populations of activated neurons. Unfortunately, the antibody that was used by Castellano et al., was discontinued, so experiments presented here were performed using a different antibody. This may suggest that TIMP2 activates c-Fos in a specific subpopulation of neurons that is not recognized as strongly by the current antibodies. The identification of the downstream c-Fos signaling pathways that may contribute to the improved cognition will be an important future direction of this research.

The IgG4 fusion to TIMP2 increased the plasma half-life of TIMP2 while still promoting improvements in cognition and synaptic density, which suggests a potential therapeutic path forward in humans. Interestingly for excitatory synaptic density, TIMP2-hIgG4 administration led to a significant increase in synapses in both the CA1 and DG, while TIMP2 only increased excitatory synapses within the DG. These findings suggest that increasing plasma half-life may lead to more widespread neuronal improvements within the hippocampus and may enhance the therapeutic benefits. Furthermore, they also suggest that the DG may be more susceptible to synaptic improvements with systemic treatment than other hippocampal brain regions. These increases in excitatory synapses are consistent with previous reports of enhanced LTP with TIMP2 treatment (Castellano et al., 2017). In addition, Ala-TIMP2 increased excitatory synapses in the DG, which suggests that the inhibitory activity of TIMP2 is not necessary for synaptic improvements.

Consistent with previous findings, neurogenesis was unchanged with TIMP2 treatment (Castellano et al., 2017). Additionally, no microglial or inflammatory markers tested in this study were modified by treatment. This suggests that the behavioral improvements in aged C57BL/6J mice following TIMP2 treatment are unlikely to be because of effects on neurogenesis or microglia activation, and instead could be because of a direct effect on neurons themselves to modulate neuronal activity via upregulation of c-Fos and excitatory synapses. Both TIMP2 and TIMP2-hIgG4 can enter the brain parenchyma when administered peripherally, which further supports direct action on neurons as a possible mechanism.

Interestingly, MMP binding to and inhibition by TIMP2 were not always correlated. The alanine inserted into TIMP2 was sufficient to significantly attenuate TIMP2-mediated MMP inhibition without affecting protein binding. In addition, the IgG4 fusion was seen to significantly attenuate TIMP2-mediated MMP binding, but not inhibition. It is likely that since TIMP2 inhibition is regulated by the N-terminus (Butler et al., 1999; Brew and Nagase, 2010) and binding is heavily dependent on its C-terminus (X. Xu et al., 2011), fusing a human IgG4Fc fragment to the C-terminus, without a linker, could also sterically hinder binding of MMPs. These results show that inhibition was not affected by IgG fusion, which may suggest that the negative impacts of steric hinderance may only be significant at small timescales. Moreover, the beneficial effects observed on cognition and neural activity appeared to be MMP independent, so any potential changes in MMP inhibitory activity with an IgG4Fc fusion are unlikely to impact improvements, as confirmed in the present study.

There are a few limitations to the results presented here. First, only male mice were used to replicate the previously reported TIMP2 findings by Castellano et al. (2017). Second, mice were single housed to prevent any complications from aggression following behavioral assays and to reduce variability within the assays. Therefore, the conclusions presented here can only be generalized to these conditions. Future studies should explore the impact of TIMP2 on female mice, especially considering recent studies highlighting that the variability in female mouse behavior may be independent of estrous cycle (Levy et al., 2023). Moreover, TIMP2 is reduced with androgens *in vitro* (Bratland et al., 2003), so it would be interesting to determine whether sex impacts TIMP2 supplementation *in vivo*.

Considering the improvements in both excitatory synapses and memory in Y-maze with Ala-TIMP2 and the fact that Ala-TIMP2 cannot inhibit MMPs at the levels found in the plasma, MMP inhibition is unlikely to be the mechanism behind these beneficial effects of TIMP2 treatment. The TIMP family of proteins has multiple protein-protein interactions that are distinct from MMP binding (Grünwald et al., 2019; Peeney et al., 2022). TIMP2 is known to have MMP-independent functions through its interactions with α 3β1-integrin (Seo et al., 2003, 2008, 2011; Vanhoutte and Heymans, 2010; Oh et al., 2004; S. H. Kim et al., 2012; Remillard et al., 2014), low-density lipoprotein receptor-related protein (LRP; Vanhoutte and Heymans, 2010), insulin-like growth factor-1 receptor (IGF-IR; Fernandez et al., 2010; Remillard et al., 2014), and vascular endothelial growth factor-A (VEGF-A; H. J. Kim et al., 2014; Remillard et al., 2014), all of which have roles in synaptic plasticity (Ivins et al., 1998; May et al., 2004; Bernard-Trifilo et al., 2005; Tillo et al., 2012; de Rossi et al., 2016; Gazit et al., 2016; Mosca et al., 2017; Lilja and Ivaska, 2018; Noriega-Prieto et al., 2021). It is possible that the cognitive improvement in aged mice with peripheral TIMP2 administration could be modulated by one of these additional interacting partners, and these questions may warrant further exploration of the interactome of TIMP2 within the CNS.

Altogether, fusion of recombinant TIMP2 to IgG4Fc was determined to extend the half-life of TIMP2 while retaining the beneficial cognitive and neuronal effects in aged C57BL/6J mice. TIMP2 has a short half-life in blood (Castellano et al., 2017), which makes its use as a therapeutic in humans challenging, but the extended half-life of TIMP2-hIgG4 may overcome these issues. In addition, experiments with the Ala-TIMP2 construct showed that MMP inhibitory activity is not essential for these beneficial outcomes. In clinical studies for cancer treatments, use of MMP inhibitors has been associated with musculoskeletal side effects (Nemunaitis et al., 1998; Wojtowicz-Praga et al., 1998; Rosemurgy et al., 1999; Tierney et al., 1999; King et al., 2003; Peterson, 2006; Fingleton, 2008), so the ability of Ala-TIMP2 to provide beneficial effects without acting on MMPs may make it a more viable approach. Together with the comprehensive assessment of the MMP inhibitory and binding impacts of these engineered proteins, these data provide important details for a therapeutic path forward for TIMP2 recombinant proteins in aging-related cognitive decline.

## Synthesis

Reviewing Editor: Michael Michaelides, NIDA-NIH Decisions are customarily a result of the Reviewing Editor and the peer reviewers coming together and discussing their recommendations until a consensus is reached. When revisions are invited, a fact-based synthesis statement explaining their decision and outlining what is needed to prepare a revision will be listed below. The following reviewer(s) agreed to reveal their identity: William Stetler-Stevenson, Baris Alten.

Reviewer 1

This manuscript reports that systemic administration of exogenous, recombinant TIMP2 or a TIMP2 fusion protein with an extended circulating half-life, TIMP2-hIgG4, results in improved cognitive function in 23 month-old, male C57BL/6J mice. Behavioral analysis included an assessment of nesting and Y-maze activities. Results demonstrate an increase in hippocampal c-Fos expression (reflected in a slight, non-statistical rise in total brain expression) and increased excitatory synaptic density. The authors also include a detailed biochemical analysis of the TIMP2, TIMP-hIgG4, and Ala-TIMP2 binding and MMP-inhibitory activities, which contributes to the overall strength of this manuscript.

The findings demonstrate that the observed effects of systemic administration of TIMP2 are independent of metalloproteinase inhibitory activity. These results are clearly in line with the emerging interest in TIMPs as direct mediators of cell behavior as recently reviewed (Grünwald, B. et al.,DOI: 10.1016/j.tcb.2018.08.006;Peeney, D. et al.,DOI: 10.1093/carcin/bgac037). Identification of the c-Fos downstream signaling pathways involved in the improved cognitive effects should be the next step.

Minor comment: Several minor grammatical errors should be corrected before publication.

Reviewer 2

• The aim of this study to create a hybrid TIMP2, which would have a longer half-life reducing the frequency of dosing for the sake of convenience and helping to translate TIMP2 from bench to bedside. Per Castellano 2017 paper, TIMP2 does have a short half life of 4.33 hours (see extended data figure 7, panel n showing first-order elimination kinetics of 64Cu-labelled TIMP2). However, TIMP2 seems to accumulate post blood-brain barrier, where radiolabeled TIMP2 can be detected at high levels (see Castellano 2017 paper stating “64Cu-radiolabelled TIMP2 injected at this dose was detected in brain at significantly higher levels than albumin control and persisted 24h after injection, while blood TIMP2 levels declined”). As TIMP2 expected to exert its after sought effects in CNS, rather than in peripheral blood; it is critical to show TIMP2-hIgG4 levels in brain as well after a single intraperitoneal administration compared to regular TIMP2. (I) Please provide data regarding TIMP2-hIgG4 levels in the brain post IP administration at physiologic doses (rather than supraphysiologic one at 1 mg/kg as shown in figure 3J). (II) In addition, please explain why the levels of TIMP2-hIgG4 1h post administration is not shown in Figure 1A and (III) explain why the dose of 250 ug/kg is used throughout the study. Seems like Castellano paper used 25-100 ug/kg. (IV) Last but not least, please provide calculations for half-life of TIMP2-hIgG4 in the blood (? vs 4.33h).

• Interestingly, 50 ug/kg of regular TIMP2 given every other that, has already been shown to improve spatial memory tasks and augment c-fos expression is Castellano 2017 study. In this study, TIMP2 was given at a dose of 250 ug/kg everyday (5 times of what was shown to be effective when given every other day). On the other hand, TIMP2-hIgG4 was given every third day. Throughout this study, there has been no significant difference between TIMP2 and TIMP2-hIgG4 on behavioral experiments, concerning for a ceiling effect with high doses of TIMP2 administration every day. I wonder, when compared with lower dose of TIMP2 (50 ug/kg) given every other day, could TIMP2-hIgG4 provide better cognitive outcomes in behavioral paradigms?

• Is there any cross-reactivity between mouse TIMP2 and human TIMP2 in the ELISA that was used for Figure 1G?

• With TIMP2 treatment, the cfos mRNA levels go up without any change in the number of cfos +ve cells in the hippocampus per Figure2A-E. Please discuss in detail that why there is a significant difference between Castellano 2017 and this study in terms of TIMP2 failing to increase cfos +ve cell count. Seems like TIMP2 consistently increased number of cfos +ve cells in Castellano paper (see figure 3, panels b and i).

• I can’t see how experiments showing absence of difference in cfos +ve cells with TIMP2 support the claim of “effects of TIMP2 and TIMP2-hIgG4 treatment in the brain are not due to large widespread impacts across multiple cell types, but rather may be specific to neurons and neuronal activity” (see page 43, lines 538-540).

• Regarding the claim “This suggests that not only did administration of human protein not increase neuroinflammation, but that the behavioral improvements in aged mice following TIMP2 treatment are unlikely to be due to effects on neuroinflammation, and instead may be due to a direct effect on neurons themselves”, I would try to soften this conclusion. There might be evidence of neuroinflammation that could have been shown biochemically (for example by measuring proinflammatory cytokines in the brain tissue itself) without changes in CD68 and Iba1. In addition, TIMP2 still can have an effect on cells other than neurons, for example astrocytes, and change how neurons behave indirectly via mechanisms other than neuroinflammation. Last but not least, just because TIMP2 is shown in brain parenchyma at high doses (Figure 3J, lines 580-581 on page 44), it does not necessarily support the notion of TIMP2 acting on neurons.

• Explain the rationale behind why PSD-95 was used as the excitatory postsynaptic marker in Figure 3, and Homer1 in Figure 6. Please repeat the experiment with PSD-95 to maintain comparability across data.

• Throughout this study, male mice were used. Traditionally, male mice have been used for behavioral experiments, especially. However, there has been a movement of including both sexes in modern behavior research to augment the generalizability of the results. There is now evidence that spontaneous behavior of female mice is not significantly affected by the estrus cycle (Levy et al, Current Biology, 2023 PMID: 36889318). I’d appreciate this being listed as a limitation of this study but it would be helpful if the most important experiment, which is the hippocampal memory dependent Y-maze with female mice. This is also something that the original Castellano paper is missing.

## Author Response

Reviewer 1 (R1) comments to author:

R1, Issue 1: These results are clearly in line with the emerging interest in TIMPs as direct mediators of cell behavior as recently reviewed (Grünwald, B. et al.,DOI: 10.1016/j.tcb.2018.08.006;Peeney, D. et al.,DOI: 10.1093/carcin/bgac037).

Response 1: We appreciate this reviewer’s comment and have included the two suggested reviews in our list of references.

R1, Issue 2: Identification of the c-Fos downstream signaling pathways involved in the improved cognitive effects should be the next step.

Response 2: We agree that the identification of the downstream signaling pathways involved in the improvement of cognition is an important next step. While outside of the scope for this manuscript, we have expanded comments addressing important next steps within the discussion section on page 49.

R1, Issue 3: Several minor grammatical errors should be corrected before publication.

Response 3: We have proofread the manuscript carefully and corrected minor grammatical errors.

Reviewer 2 (R1) comments to author:

R2, Issue 1: As TIMP2 expected to exert its after sought effects in CNS, rather than in peripheral blood; it is critical to show TIMP2-hIgG4 levels in brain as well after a single intraperitoneal administration compared to regular TIMP2. (I) Please provide data regarding TIMP2-hIgG4 levels in the brain post IP administration at physiologic doses (rather than supraphysiologic one at 1 mg/kg as shown in figure 3J). (II) In addition, please explain why the levels of TIMP2- hIgG4 1h post administration is not shown in Figure 1A and (III) explain why the dose of 250 ug/kg is used throughout the study. Seems like Castellano paper used 25-100 ug/kg. (IV) Last but not least, please provide calculations for half-life of TIMP2-hIgG4 in the blood (? vs 4.33h).

Response 1:

(I) To address this reviewer’s question, we measured the levels of TIMP2 in brain lysate of mice treated with Vehicle, TIMP2, or TIMP2-hIgG4 at physiologic doses (250 μg/kg) by ELISA. Using this technique, we were unable to detect any appreciable TIMP2 in the brain of either TIMP2 or TIMP2-hIgG4 treated mice, Figure 1. This result was unsurprising based on the data presented in Castellano et al. We did not anticipate TIMP2 levels in the brain to be high enough with a 250 μg/kg dose to detect using our ELISA based approach. The lower limit of detection for this ELISA is 31.2 pg/mL and with a 1:3 dilution factor for brain lysate, the lowest detectable 3 (II) level corresponds to 10.4 pg/mL. Castellano et al. utilized autoradiography of 64Cu-labled TIMP2, which provides a significantly higher sensitivity than a standard ELISA based approach. Therefore, we chose to test brain penetrance using a 1 mg/kg dose in the manuscript. Future studies could explore more sensitive techniques, such as micro-autoradiography (mARG), to both detect the level of TIMP2 in the brain, as well as the localization within the brain parenchyma. However, this was beyond the scope of the work presented in this manuscript. Figure 1: Brain Levels of TIMP2 Following Dosing at Physiological Levels (250 μg/kg) A, Human TIMP2 in mouse hemibrain after administration of protein (250 μg/kg). n = 3 mice per time point. B, The same data presented in A, combined with the data from the manuscript Figure 3J, to show that the levels of TIMP2 in the brain following dosing at physiological levels is below the level of detection in our ELISA. (III) For this experiment, TIMP2-hIgG4 was not measured at 1 hour post-administration.

Since the addition of IgG to protein constructs is a well-known strategy for extending half-life (Strohl, 2018), we chose to start our PK measurement at 2 hours post-dose. Strohl, R. W. (2018). Current progress in innovative engineered antibodies, 9(1), 86-120. https://doi.org/10.1007/s13238-017-0457-8 (IV) The administration of 50 μg/kg of TIMP2 was not efficacious in our hands and the 250 μg/kg dose for TIMP2 was chosen based on two pilot experiments indicating that a higher dose may be necessary for improved cognition. These pilot experiments were not fully powered and therefore we decided not to include them in the manuscript. If the reviewers and editors deem appropriate, we can incorporate the following two figures into the manuscript. Figure 2 below shows the Y-maze data from an experiment replicating the dosing paradigm published in Castellano et al. In this test, aged mice treated with 0246 0.000 0.001 0.002 0.003 0.004 Human TIMP2 (nM) Hours Post Administration TIMP2 0.25mg/kg TIMP2-hIgG4 0.25mg/kg Vehicle (0.25mg/kg) 0246 0.000 0.005 0.010 0.015 0.020 Human TIMP2 (nM) Vehicle (1mg/kg) TIMP2 1mg/kg TIMP2-hIgG4 1mg/kg Hours Post Administration * * TIMP2 0.25mg/kg TIMP2-hIgG4 0.25mg/kg Vehicle (0.25mg/kg) A B 4 recombinant TIMP2 purchased from R&D Systems failed to show a preference for the novel arm in the Y-maze. Young mice were used as a positive control in this experiment.

The experiment was repeated with recombinant TIMP2 protein purified in house. Figure 3 below shows the Y-maze data from this experiment where mice were dosed with either 50 μg/kg or 250 μg/kg TIMP2. Aged mice treated with the lower dose failed to show a preference for the novel arm, while mice treated with the higher dose displayed a trend (n=6).

The difference between Castellano et al. and our findings may be due to variances between cohorts of aged mice. Castellano et al. utilized aged mice from the National Institutes of Health aging colony, while mice used for these studies were purchased from Jackson Labs. Previous groups have reported genetic and behavioral differences between these two colonies (Bryant et al., 2009). Additionally, the differences could be due to batch or vendor differences in the recombinant TIMP2 proteins. We provide extensive characterization of the in house purified TIMP2 to verify purity and activity. Bryant, C. D., Zhang, N. N., Sokoloff, G., Fanselow, M. S., Ennes, H. S., Palmer, A. A., & McRoberts, J. A. (2009, Jul 11). Behavioral Differences among C57BL/6 Substrains: Implications for Transgenic and Knockout Studies. Journal of Neurogenetics, 22(4): 315-331. https://doi.org/10.1080/01677060802357388 Figure 2: Y-maze Following Treatment of Aged C57BL/6 Mice with the Previously Published Dosing Paradigm 5

Y-maze performance after administration of TIMP2 R&D (50 μg/kg) every other day for 4 weeks in 18-month-old C57BL/6 mice. n = 14 mice per treatment group. An additional 6 young mice aged 3 months were used as a positive control. Figure 3: Y-maze Following Treatment of Aged C57BL/6 Mice with Escalating TIMP2 Dose Y-maze performance after administration of recombinant TIMP2 purified in house (50 μg/kg or 250 μg/kg) every day for 3.5 weeks in 18-month-old C57BL/6 mice. n = 6-12 mice per treatment group. (V) A non-linear regression analysis was performed to fit a straight line through the data from Figure 1A using the Prism 8 software to calculate the half-life. Using this calculation, the half-life for TIMP2 is estimated to be 1.8 hours and the half-life for TIMP2-hIgG4 is 16.9 hours. As the experiment wasn’t intended for a calculation of half-life, this calculation provides a rough estimate. To properly calculate a half-life, the experiment would need to be repeated with additional timepoints on the shorter end of the timescale. However, since our estimate of half-life is within the same range as Castellano et al. (4.33 hours), we hope the reviewer agrees that this calculation is sufficient and does not justify the additional use of mice to further refine the value. R2, Issue 2: Interestingly, 50 ug/kg of regular TIMP2 given every other that, has already been shown to improve spatial memory tasks and augment c-fos expression is Castellano 2017 study.

In this study, TIMP2 was given at a dose of 250 ug/kg everyday (5 times of what was shown to be effective when given every other day). On the other hand, TIMP2-hIgG4 was given every third day. Throughout this study, there has been no significant difference between TIMP2 and TIMP2-hIgG4 on behavioral experiments, concerning for a ceiling effect with high doses of 6

TIMP2 administration every day. I wonder, when compared with lower dose of TIMP2 (50 ug/kg) given every other day, could TIMP2-hIgG4 provide better cognitive outcomes in behavioral paradigms?

Response 2:

The reviewer raises an important series of questions in the last two comments regarding the determination of an effective dose of TIMP2. To translate TIMP2 into possible future therapeutics, it will be important for future studies and additional labs to explore efficacious doses, dosing paradigms, and TIMP2 constructs. As outlined in Response 1, the administration of 50 μg/kg of TIMP2 was not efficacious in our hands and the 250 μg/kg dose for TIMP2 was chosen based on pilot experiments indicating that a higher dose may be necessary for improved cognition. We chose to dose TIMP2-hIgG4 every third day to achieve a similar level of circulating TIMP2 in the plasma based on our PK curves (Figure 1A) and half-life calculations (response 1).

R2, Issue 3: Is there any cross-reactivity between mouse TIMP2 and human TIMP2 in the ELISA that was used for Figure 1G?

Response 3: To measure mouse TIMP2, we used an ELISA from R&D Systems (Cat #: DY6304; https://www.rndsystems.com/products/mouse-timp-2-duoset-elisa_dy6304-05). The product datasheet states there is 0.2% cross-reactivity when testing a sample containing 25 ng/mL of recombinant human TIMP2. Additionally, the lack of cross-reactivity with human TIMP2 in the mouse plasma is evidenced by the low background in our ELISA results from mouse plasma with 50 pg/mL of spiked in recombinant human TIMP2 protein seen below, Figure 4. Figure 4: Terminal Plasma from Aged Mice Treated with Vehicle or TIMP2 Endogenous mouse TIMP2 protein in plasma after 4 weeks of chronic administration of human TIMP2 protein. n = 10-13 per group. Vehicle, TIMP2, and TIMP2-hIgG4 data is presented in the manuscript Figure 1G. As a control, 50 pg/mL human TIMP2 protein 7 was spiked into control mouse plasma and there was no additional signal detected, indicating little cross-reactivity of the mouse ELISA with the human protein. The data presented here does not take into account the dilution factor in the Vehicle, TIMP2, and TIMP2-hIgG4 groups to allow for better visualization of the spike-in sample. To measure human TIMP2, we used an ELISA from R&D Systems (Cat #: DY971; https://www.rndsystems.com/products/human-timp-2-duoset-elisa_dy971). The product datasheet states there is no cross-reactivity with mouse TIMP2 assayed at 50 ng/mL.

Additionally, the lack of cross-reactivity with mouse TIMP2 in the plasma is evidenced by the low background in our ELISA results from vehicle-treated mice seen below, Figure 5. Figure 5: Terminal Plasma from Aged Mice Treated with Vehicle or TIMP2 Human TIMP2 in mouse plasma after administration of TIMP2 (250 μg/kg) every day for 4 weeks in 21-month-old C57BL/6 mice. n = 17-18 mice per treatment group. R2, Issue 4: With TIMP2 treatment, the cfos mRNA levels go up without any change in the number of cfos +ve cells in the hippocampus per Figure2A-E. Please discuss in detail that why there is a significant difference between Castellano 2017 and this study in terms of TIMP2 failing to increase cfos +ve cell count. Seems like TIMP2 consistently increased number of cfos +ve cells in Castellano paper (see figure 3, panels b and i).

Response 4: We were also surprised we were unable to replicate the c-Fos histological findings from Castellano et al. However, we believe that reporting the difference between the two labs is Vehicle TIMP2 0 1 2 3 4 TIMP2 (nM) 8 important and highlights one of the challenges of replication studies. In the first paragraph in the discussion section on pages 48-49, we outline some of the possible differences between Castellano 2017 and our studies. We have copied this text below. Due to the discontinuation of the antibody used in Castellano et al., we were unable to fully replicate the previously published protocol and unfortunately will never be able to fully answer why we observed a different result. First, it is possible that although changes in gene expression of cfos were detectable in our study, the time point was not optimized to detect changes by histology. Furthermore, it is likely that different c-Fos antibodies recognize different populations of activated neurons. Unfortunately, the antibody that was used by Castellano et al. was discontinued, so histological readouts and the iDISCO experiment were performed using a different antibody. This may suggest that TIMP2 activates c-Fos in a specific sub-population of neurons that is not recognized as strongly by the current antibodies. To further expand on the possibility that different c-Fos antibodies recognize different populations of activated neurons, we tested 3 commercially available c-Fos antibodies, Figure 6.

Unfortunately, each of these antibodies was purified from rabbits, so we were unable to stain for them within the same section. However, when looking at the representative images, one can appreciate the large differences in the number of labeled cells in the dentate gyrus. For our studies, we proceeded with the Cell Signaling Technology antibody due to the high contrast between the positive cells and background. Figure 6: Representative c-Fos Images of the Dentate Gyrus

To determine if different c-Fos antibodies could stain separate populations of activated neurons, sections were stained from a control, untreated mouse and a mouse that underwent a foot shock 90 minutes prior to sacrifice as a positive control. Each antibody produced a different pattern and number of positive cells. 9 R2, Issue 5: I can’t see how experiments showing absence of difference in cfos +ve cells with TIMP2 support the claim of “effects of TIMP2 and TIMP2-hIgG4 treatment in the brain are not due to large widespread impacts across multiple cell types, but rather may be specific to neurons and neuronal activity” (see page 43, lines 538-540). Regarding the claim “This suggests that not only did administration of human protein not increase neuroinflammation, but that the behavioral improvements in aged mice following TIMP2 treatment are unlikely to be due to effects on neuroinflammation, and instead may be due to a direct effect on neurons themselves”, I would try to soften this conclusion. There might be evidence of neuroinflammation that could have been shown biochemically (for example by measuring proinflammatory cytokines in the brain tissue itself) without changes in CD68 and Iba1. In addition, TIMP2 still can have an effect on cells other than neurons, for example astrocytes, and change how neurons behave indirectly via mechanisms other than neuroinflammation. Last but not least, just because TIMP2 is shown in brain parenchyma at high doses (Figure 3J, lines 580-581 on page 44), it does not necessarily support the notion of TIMP2 acting on neurons.

Response 5: The reviewer is correct that we do not provide any biochemical data on the impact of TIMP2 treatment on neuroinflammation. It is possible that TIMP2 could still influence microglia and/or astrocytes, which indirectly impact the neurons. We have softened the language surrounding this conclusion to reflect this on pages 43-44. R2, Issue 6: Explain the rationale behind why PSD-95 was used as the excitatory postsynaptic marker in Figure 3, and Homer1 in Figure 6. Please repeat the experiment with PSD-95 to maintain comparability across data.

Response 6: Unfortunately, the PSD-95 antibody lot used for Figure 3 is no longer available and the new lot does not perform similarly. The PSD-95 antibody issue has simultaneously been identified by other labs (personal communication) and is reflected in modification of protocols in published work (Dejanovic et al., 2018), which now uses the Homer1 antibody to stain for the postsynaptic density as a replacement for the poor performing PSD-95 antibody. Dejanovic, B., Huntley, M. A., De Maziere, A., Meilandt, W. J., Wu, T., Srinivasan, K., Jiang, Z., Gandham, V., Friedman, B. A., Ngu, H., Foreman, O., Carano, R. A. D., Chih, B., Klumperman, J., Bakalarski, C., Hanson, J. E., & Sheng, M. (2018, Dec 19). Changes in the Synaptic Proteome in Tauopathy and Rescue of Tau-Induced Synapse Loss by C1q Antibodies. Neuron, 100(6), 1322-1336 e1327. https://doi.org/10.1016/j.neuron.2018.10.014 R2, Issue 7: Throughout this study, male mice were used. Traditionally, male mice have been used for behavioral experiments, especially. However, there has been a movement of including both sexes in modern behavior research to augment the generalizability of the results. There is now evidence that spontaneous behavior of female mice is not significantly affected by the estrus cycle (Levy et al, Current Biology, 2023 PMID: 36889318). I’d appreciate this being listed as a limitation of this study but it would be helpful if the most important experiment, which is the hippocampal memory dependent Y-maze with female mice. This is also something that the original Castellano paper is missing. 10

Response 7: We agree with this reviewer’s comment that a limitation of this study is the utilization of only male mice. We have expanded this comment within our discussion section and included the suggested citation, now on pages 50-51.
